# Detailed characterization of SARS-CoV-2-specific T and B cells after infection or heterologous vaccination

**DOI:** 10.3389/fimmu.2023.1123724

**Published:** 2023-02-09

**Authors:** Domenico Lo Tartaro, Annamaria Paolini, Marco Mattioli, Julian Swatler, Anita Neroni, Rebecca Borella, Elena Santacroce, Alessia Di Nella, Licia Gozzi, Stefano Busani, Michela Cuccorese, Tommaso Trenti, Marianna Meschiari, Giovanni Guaraldi, Massimo Girardis, Cristina Mussini, Katarzyna Piwocka, Lara Gibellini, Andrea Cossarizza, Sara De Biasi

**Affiliations:** ^1^ Department of Medical and Surgical Sciences for Children and Adults, University of Modena and Reggio Emilia School of Medicine, Modena, Italy; ^2^ Laboratory of Cytometry, Nencki Institute of Experimental Biology, Warsaw, Poland; ^3^ Infectious Diseases Clinics, Azienda Ospedaliero-Universitaria (AOU) Policlinico di Modena, Modena, Italy; ^4^ Department of Surgery, Medicine, Dentistry and Morphological Sciences, University of Modena and Reggio Emilia, Modena, Italy; ^5^ Department of Anesthesia and Intensive Care, Azienda Ospedaliero-Universitaria (AOU) Policlinico and University of Modena and Reggio Emilia, Modena, Italy; ^6^ Department of Laboratory Medicine and Pathology, Diagnostic Hematology and Clinical Genomics, Azienda Unità Sanitaria Locale AUSL/AOU Policlinico, Modena, Italy; ^7^ National Institute for Cardiovascular Research, Bologna, Italy

**Keywords:** SARS-CoV-2, antigen-specific response, polyfunctionality, T cells, B cells, cytokine

## Abstract

The formation of a robust long-term antigen (Ag)-specific memory, both humoral and cell-mediated, is created following severe acute respiratory syndrome coronavirus 2 (SARS-CoV-2) infection or vaccination. Here, by using polychromatic flow cytometry and complex data analyses, we deeply investigated the magnitude, phenotype, and functionality of SARS-CoV-2-specific immune memory in two groups of healthy subjects after heterologous vaccination compared to a group of subjects who recovered from SARS-CoV-2 infection. We find that coronavirus disease 2019 (COVID-19) recovered patients show different long-term immunological profiles compared to those of donors who had been vaccinated with three doses. Vaccinated individuals display a skewed T helper (Th)1 Ag-specific T cell polarization and a higher percentage of Ag-specific and activated memory B cells expressing immunoglobulin (Ig)G compared to those of patients who recovered from severe COVID-19. Different polyfunctional properties characterize the two groups: recovered individuals show higher percentages of CD4^+^ T cells producing one or two cytokines simultaneously, while the vaccinated are distinguished by highly polyfunctional populations able to release four molecules, namely, CD107a, interferon (IFN)-γ, tumor necrosis factor (TNF), and interleukin (IL)-2. These data suggest that functional and phenotypic properties of SARS-CoV-2 adaptive immunity differ in recovered COVID-19 individuals and vaccinated ones.

## Introduction

Memory is the main characteristic of the immune system, being at the basis of its efficacy and functionality, and indeed the activation of secondary response is the crucial strategy utilized by vaccination. Natural infection and vaccines induce the formation and subsequent expansion of antigen (Ag)-specific cells that can block pathogens as soon as they try to invade the host. The creation of a pool of long-living memory T and B cells able to respond to future *stimuli* is crucial for vaccine efficacy, as well as the plasma level of antibodies (1).

During natural infection, typically after a couple of weeks, the magnitude of the severe acute respiratory syndrome coronavirus 2 (SARS-CoV-2)-specific CD4^+^ and CD8^+^ memory T-cell response peaks at the maximum and is of the order of 0.5% and 0.2% of the repertoire, respectively (2). CD4^+^ T cells display a memory profile (including a specific subset formed by stem cell memory) and are able to produce high levels of both IL-2 and T helper (Th)1 cytokines (3–5). CD4^+^ T-cell response is greater than the CD8^+^ counterpart (2). Robust immunity is certainly maintained by more than 6 months, but the duration of SARS-CoV-2-specific T cells could depend also on the clinical severity of the initial infection (6). Long-lived T-cell responses and efficient response to SARS-CoV-2 are characterized by a CD45RA^+^ effector-memory phenotype and a potent activation of the interferon (IFN) transcriptomic signature whose magnitude is largely due to the genetic background of the host (7–10).

Regarding B-cell response, which is highly altered during coronavirus disease 2019 (COVID-19) (11), in the plasma of most individuals, anti-SARS-CoV-2 antibodies (Abs) persist for more than 6 months after primary infection, but some patients rapidly lose their specific Abs (6, 12, 13). However, specific memory B cells (MBCs) predominantly express immunoglobulin (Ig)M^+^ or IgG1^+^ and rise until 150 days after infection (14), regardless of age (15). Receptor-binding domain (RBD)-specific IgG^+^ MBCs are predominantly CD27^+^, and their number significantly correlates with circulating follicular helper T-cell numbers (14).

Vaccination against SARS-CoV-2 induces a robust specific immune response. CD8^+^ T-cell response can be detected as early as 11 days after the first vaccination (16), and such cells can recognize immunodominant peptides from ORF1ab (17). Two-dose vaccination with BNT162b2 leads to strong generation of virus-specific CD4^+^ T-cell responses with a Th1 profile, and it is detectable 6 months after vaccination (18–21). Spike-specific antibodies peak after 7 days, and titers and *Angiotensin-converting enzyme 2* (ACE2)/RBD binding-inhibiting activity is still observed after 6 months, despite a progressive decline over time. Concomitant to antibody reduction, spike-specific MBCs, mostly switched to IgG, increase and persist 6 months after vaccination (22). T-cell responses after vaccination are of similar magnitude to those seen after natural infection, although they seem to be more differentiated with the presence of T stem cell memory (T_SCM_) subsets (23). An adenovirus-based vaccine generates a higher magnitude of spike-specific T cells (24, 25), while mRNA vaccines develop higher antibody titers. For this reason, heterologous vaccines have been used in clinical practice (26, 27).

As vaccination and natural infection increase across the world, there is growing interest in predicting the risk of primary infection or reinfection. Observational and limited comparison between natural and vaccine-induced immunity showed that the protection against SARS-CoV-2 infection was significantly higher in COVID-19 recovered individuals if compared to that of those vaccinated who additionally received a booster vaccine (28). Antibodies decline more rapidly following vaccination in naive individuals than those in individuals who have recovered from COVID-19, but they display the same frequencies of spike-specific B and CD4^+^ T cells at 8 months after vaccination (29). However, besides the magnitude of the spike-specific antibody response or neutralizing titer, the percentage, phenotypic identity, and functional profile of specific cellular immune responses have not been taken into account as immune correlates of protection.

Here, by using high-parameter polychromatic flow cytometry and sophisticated data analyses, we deeply investigated the magnitude, phenotype, and functionality of SARS-CoV-2–specific immune memory in two groups of healthy subjects after heterologous vaccination compared to those of a group of subjects who recovered from SARS-CoV-2 infection.

## Results

### Study design

Three groups of donors were enrolled in this study. The first one was composed of nine COVID-19 recovered patients (hereafter called REC; mean age of 35.1 ± 11.1 years), with a mean of 131.1 days (range 64–165 days) from last infection during follow-up visits at the Infectious Diseases Clinics of the Azienda Ospedaliero-Universitaria Policlinico di Modena. All REC had symptoms consistent with COVID-19 and positive PCR-based testing for SARS-CoV-2 over the period of March 2020–August 2020. Within this group, four patients were classified as severe (35.3 ± 5.68 years) while five patients were moderate (35.0 ± 7.4) according to World Health Organization guidelines (30). Given that there were no differences between moderate and severe recovered individuals and their low number, they were considered as a unique group for the statistical analysis. Twenty-three vaccinated donors were enrolled in this study, and they were divided into two groups: one was composed of 11 donors with a mean of 31.1 days (range 30–35 days) after the third dose of SARS-CoV-2 vaccine (hereafter defined MIX; 27.0 ± 4.5 years); these subjects were vaccinated with three different vaccines (first dose: ChAdOx1; second dose: BNT162b2; third dose: mRNA-1273). The second group was composed of 12 donors with a mean of 33.9 days (range 26–44 days, hereafter defined RNA; 35.3 ± 11.3 years) after being vaccinated with two different RNA vaccines (first and second doses: BNT162b2; third dose: mRNA-1273). Each participant, including healthy donors, provided informed consent according to the Helsinki Declaration, and all uses of human material have been approved by the local Ethics Committee (Comitato Etico dell’Area Vasta Emilia Nord, protocol number 177/2020, 11 March 2020) and by the University Hospital Committee (Direzione Sanitaria dell’Azienda Ospedaliero Universitaria di Modena, protocol number 7531, 11 March 2020). The clinical characteristics of all participants are reported in **Table 1** and in the *Methods* section.

**Table 1:** Demographic and clinical characteristics of COVID-19 recovered patients and vaccinated donors.

Variable	REC (n=9)	MIX (n=11)	RNA (n=12)	p-value (REC*vs*MIX)	p-value (REC*vs*RNA)	p-value (MIX*vs*RNA)
Demographic characteristics						
Age (mean years, range)^1^	35.1 (22.0-41.0)	27.0 (22.0-39.0)	35.3 (23.0-62.0)	0.0177	ns	ns
Sex (Male, %)^2^	88.9	27.3	45.5	0.0098	ns	ns
Race/Ethnicity						
White: Non-Hispanic or Latino (%)^3^	66.7	100	100	ns	ns	ns
White: Hispanic or Latino (%)^3^	11.1	0	0	/	/	/
Black (%)^3^	22.2	0	0	/	/	/
Hospitalization status						
Never hospitalized (%)	11.1	/	/	/	/	/
Hospitalized (%)	88.9	/	/	/	/	/
Days of hospitalization (mean days, range)	11.6 (4.0-17.0)	/	/	/	/	/
Sample Collection						
Sample Collection Dates	March 2020- August 2020	December 2021-January 2022	December 2021- January 2022	/	/	/
Days post symptom onset or third dose vaccine (mean days, range) ^1^	131.1 (64.0-165.0)	31.8 (30.0-35.0)	33.9 (26.0-44.0)	0.0004	0.0009	ns
Disease Severity						
Moderate (%)	55.6% (5/9)	/	/	/	/	/
Severe (%)	44.4% (4/9)	/	/	/	/	/
Vaccine type						
First dose	/	ChAdOx1	BNT162b2	/	/	/
Second dose	/	BNT162b2	BNT162b2	/	/	/
Third dose	/	mRNA-1273	mRNA-1273	/	/	/
Detection of SARS-CoV-2 IgG						
IgG, Index mean value AU/mL (± SD)^1^	1,344.2 (±663.4)	12,205.4 (±9,457.4)	6,422.1 (±2,496.1)	0.0001	0.0011	ns

^1^Kruskal-Wallis test with Original FDR methods of Benjamini and Hochberg;

^2^Fisher's exact test;

^3^Chi-squared test;

ns, not significant; SD, standard deviation; AU, arbitrary unit.

### MIX showed a skewed Th1 Ag-specific CD4^+^ T-cell polarization compared to that of recovered ones

To investigate the percentage of Ag-specific T cells, we used T-cell receptor (TCR)-dependent activation-induced marker (AIM) assays to identify and quantify SARS-CoV-2-specific CD4^+^ T cells (31–34). We stimulated peripheral blood mononuclear cells (PBMCs) from nine REC patients and 11 MIX and 12 RNA donors overnight with 15-mer peptides with 11-amino acid overlap, covering the complete sequence of Wuhan SARS-CoV-2 spike glycoprotein (see *Methods* for details).

The phenotype of Ag-specific T cells (i.e., those CD137^+^CD69^+^) within CD4^+^ T cells, hereafter termed Ag^+^CD4^+^ T cells, was first analyzed by manual gating and compared with the non-Ag-specific CD4^+^ T cell counterparts (CD137^−^CD69^−^, hereafter called Ag^−^CD4^+^). Ag^+^CD4^+^ T cells showed different cell subset distributions (in terms of the expression of differentiation markers such as CD45RA, CCR7, CD28, and CD95) and Th cell polarization (evaluated by the expression of CCR6 and CXCR3). Ag^+^CD4^+^ T displayed a low percentage of naive (N, CD45RA^+^CCR7^+^CD28^+^CD95^−^) and higher frequencies of memory compartment such as central memory (CM; CD45RA^−^CCR7^+^CD28^+^CD95^+^), transitional memory (TM; CD45RA^−^CCR7^−^CD28^+^CD95^+^), effector memory (EM; CD45RA^−^CCR7^−^CD28^−^CD95^+^), and T_SCM_ (CD45RA^+^CCR7^+^CD28^+^CD95^+^) and a similar percentage of terminally differentiated effector memory (EMRA; CD45RA^+^CCR7^-^CD28^-^CD95^+^) (**Supplementary Figures S1A, B**). Considering T-cell polarization, in comparison with Ag^−^CD4^+^ T cells, those Ag-specific displayed a higher percentage of Th1 (CXCR3^+^CCR6^−^), Th17 (CXCR3^−^CCR6^+^), and Th1/Th17 (CXCR3+CCR6+) and a lower percentage of Th0/Th2 (CXCR3^−^CCR6^−^) (**Supplementary Figure S1C**).

**Figure 1:**
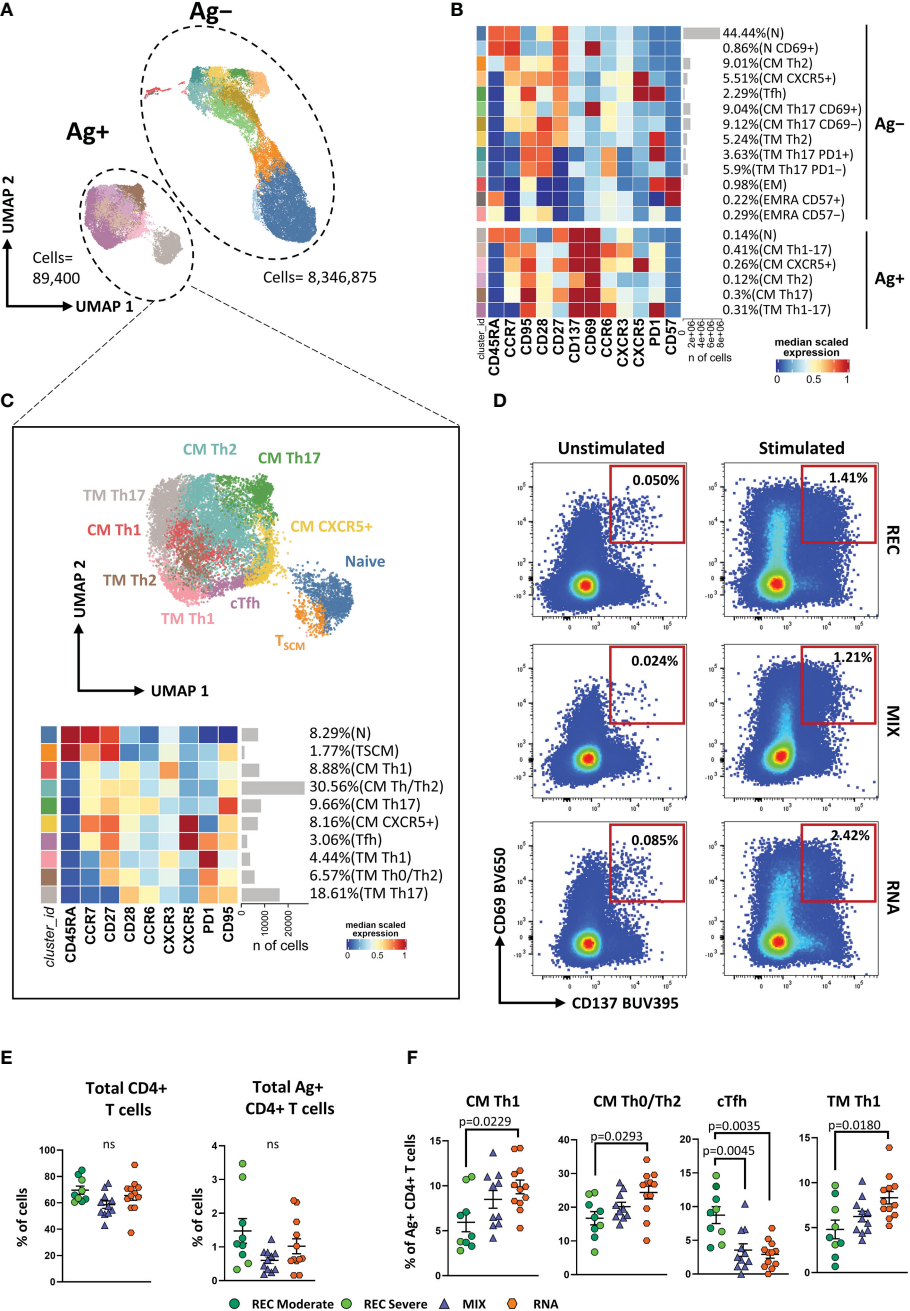
Immune phenotyping of antigen-specific CD4^+^ T cells. **(A)** Uniform Manifold Approximation and Projection (UMAP) plot shows the 2D spatial distribution of 8,436,275 cells from nine donors who recovered from SARS-CoV-2 infection (REC, severe = 4 and moderate = 5) and 23 vaccinated donors (MIX = 11 and RNA = 12) embedded with FlowSOM clusters. Ag^+^, antigen-specific CD4^+^ T cells; Ag^−^, non-antigen-specific CD4^+^ T cells. **(B)** Heatmap of the median marker intensities of the 12 lineage markers across the 19 cell populations obtained with FlowSOM algorithm after the manual metacluster merging. The colors of cluster_id column correspond to the colors used to label the UMAP plot clusters. The color in the heatmap is referred to the median of the *arcsinh* marker expression (0–1 scaled) calculated over cells from all of the samples. Blue represents lower expression, while red represents higher expression. Light gray bar along the rows (clusters) and values in brackets indicate the relative sizes of the clusters. N, naive; T_SCM_, T stem cell memory; CM, central memory; TM, transitional memory; EM, effector memory; EMRA, effector memory reexpressing the CD45RA; cTfh, circulating T follicular helper cells. The black bar on the right is used to group Ag^+^ or Ag^−^ subpopulations. **(C)** UMAP and heatmap visualization of 10 manually merged antigen-specific CD4^+^ T-cell clusters. **(D)** Representative dot plots showing manual gating analysis of Ag-specific (CD137^+^CD69^+^) CD4^+^ T cells after overnight stimulation with spike protein compared to unstimulated control [activation-induced marker assay (AIM assay)]. Numbers in the dot plots indicate the percentage of cells identified by manual gating. **(E)** Dot plots show the total percentage of antigen-specific CD4^+^ T cells. Kruskal–Wallis test with Benjamini–Hochberg correction for multiple comparisons was used to test the differences among the three groups. **(F)** Dot plots show the cell percentage of the antigen-specific CD4^+^ T cells. The central bar represents the mean ± SEM. Generalized linear mixed model (GLMM) test was used for the statistical analysis. Adjusted P-values are reported in the figure. ns, not significant.

To gain a more detailed overview on the differentiation status and Th-polarization, we took advantage of unsupervised FlowSOM clustering. This analysis revealed a total of 19 clusters, and within these, six clusters represented SARS-CoV-2-reactive CD4^+^ T cells expressing CD69 and CD137 (**Figures 1A, B**; **Supplementary Figure S2**).

The frequencies of the different clusters of T cells within Ag^−^CD4^+^ T cells were similar in the three groups of individuals as shown in **Supplementary Figure S3**. We focused our attention on Ag^+^CD4^+^ T cells that were selected and reclustered. We obtained 10 clusters, representing different subpopulations of Ag^+^ T cells. We found naive T cells that were defined as CD45RA^+^CCR7^+^CD27^+^ CD95^−^, T_SCM_ as CD45RA^+^CCR7^+^CD27^+^CD95^+^, CM Th1 as CCR7^+^CD45RA^−^CCR6^−^CXCR3^+^ (CM Th1), CM Th0/Th2 as CCR7^+^CD45RA^−^CCR6^−^CXCR3^−^, CM Th17 as CCR7^+^CD45RA^−^CCR6^+^CXCR3^−^, CM CXCR5^+^ as CCR7^+^CD45RA^−^CXCR5^+^PD-1^−^, circulating T follicular helper as CCR7^+^CD45RA^−^CXCR5^+^PD-1^+^ (cTfh), TM Th1 as CCR7^−^CD45RA^−^CD28^+^CXCR3^+^ (TM Th1), TM Th0/Th2 as CCR7^−^CD45RA^−^CD28^+^CCR6^−^CXCR3^−^, and TM Th17 as CCR7^−^CD45RA^−^CD28^+^CCR6^+^CXCR3^−^(**Figure 1C**).

The percentage of total CD4^+^ and Ag^+^ CD4^+^ T cells was similar among the three groups (**Figures 1D, E**). Despite that, within the latter, we observed a different distribution of the populations among REC and vaccinated groups (both MIX and RNA). RNA displayed higher percentages of CM Th1, CM Th0/Th2, and TM Th1 if compared to those in REC subjects. Moreover, both MIX and RNA showed a lower percentage of cTfh cells (**Figure 1F**). No differences were found between Ag^+^CD4^+^ T-cell clusters of MIX and RNA. Similar percentages of all other clusters were present in REC, MIX, and RNA subjects (**Supplementary Figure S4**).

### Vaccinated individuals showed a higher percentage of Tc1-like Ag-specific CD8^+^ T cells compared to that of recovered subjects

The AIM assay was used for CD8^+^ T-cell analysis to identify and quantify SARS-CoV-2-specific (see *Methods*). We first manually gated different subpopulations of T cells on the basis of differentiation markers and cytotoxic-polarization markers (Tc-polarization). We observed that Ag^+^CD8^+^ T cells, if compared to Ag^−^CD8^+^ T lymphocytes, displayed lower percentages of N and higher percentages of T_SCM_, CM, and TM; similar percentages of both EM and EMRA were found (**Supplementary Figures S5A, B**). In terms of Tc-polarization, similar percentages of Tc1 cells were found within Ag^+^ and Ag^−^CD8^+^ T cells. However, Ag^+^CD8^+^ T cells were characterized by higher percentages of both Tc17 and Tc1/Tc17 and lower percentages of Tc0/Tc2 (**Supplementary Figure S5C**).

**Figure 2:**
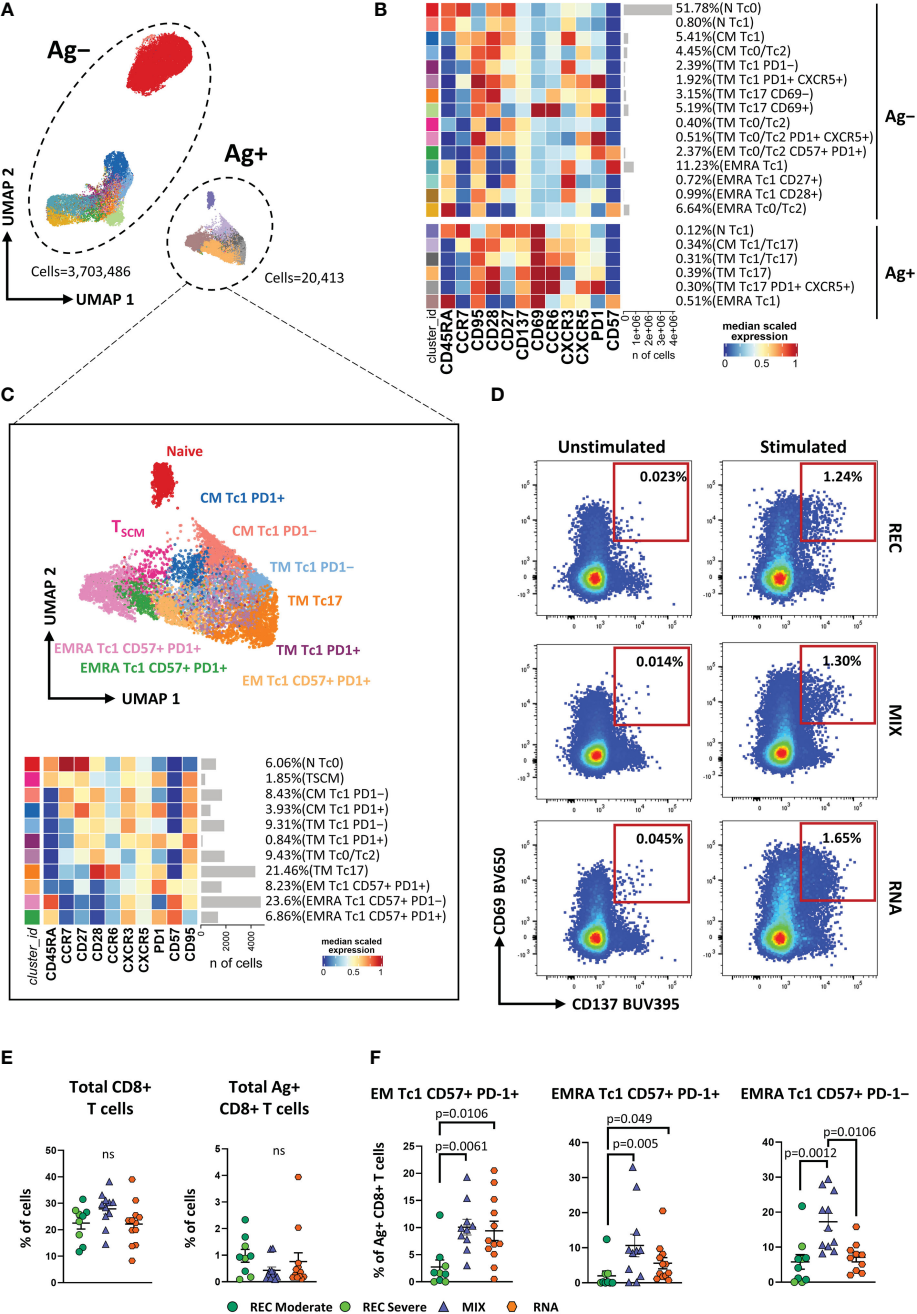
Immune phenotyping of antigen-specific CD8^+^ T cells. **(A)** UMAP plot shows the 2D spatial distribution of 3,723,899 cells from nine donors who recovered from SARS-CoV-2 infection (REC, severe = 4 and moderate = 5) and 23 vaccinated donors (MIX = 11 and RNA = 12) embedded with FlowSOM clusters. Ag^+^, antigen-specific CD8^+^ T cells; Ag^−^, non-antigen-specific CD8^+^ T cells. **(B)** Heatmap of the median marker intensities of the 12 lineage markers across the 21 cell populations obtained with FlowSOM algorithm after the manual metacluster merging. The colors of cluster_id column correspond to the colors used to label the UMAP plot clusters. The color in the heatmap is referred to the median of the *arcsinh* marker expression (0–1 scaled) calculated over cells from all of the samples. Blue represents lower expression, while red represents higher expression. Light gray bar along the rows (clusters) and values in brackets indicate the relative sizes of the clusters. N, naive; CM, central memory; TM, transitional memory; EM, effector memory; EMRA, effector memory reexpressing the CD45RA. The black bar on the right is used to group Ag^+^ or Ag^−^ subpopulations. **(C)** Uniform Manifold Approximation and Projection UMAP and heatmap visualization of 11 manually merged antigen-specific CD8^+^ T cell clusters. **(D)** Representative dot plots showing manual gating analysis of Ag-specific (CD137^+^CD69^+^) CD8^+^ T cells after overnight stimulation with spike protein compared to unstimulated control [activation-induced marker assay (AIM assay)]. Numbers in the dot plots indicate the percentage of cells identified by manual gating. **(E)** Dot plots show the total percentage of antigen-specific CD8^+^ T cells. Kruskal–Wallis test with Benjamini–Hochberg correction for multiple comparisons was used to test the differences among the three groups. **(F)** Dot plots show the relative cell percentage of the antigen-specific CD8^+^ T-cell clusters of nine donors who recovered from SARS-CoV-2 infection (REC, severe = 4 and moderate = 5) and 23 vaccinated donors (MIX = 11 and RNA = 12). The central bar represents the mean ± SEM. Generalized linear mixed model GLMM test was used for the statistical analysis. Adjusted P-values are reported in the figure. ns, not significant.

As for CD4^+^ T-cell analysis, we applied unsupervised analysis and found 21 clusters, of which six were SARS-CoV-2-reactive CD8^+^ T cells (**Figures 2A, B**; **Supplementary Figure S6A**). Considering Ag^−^CD8^+^ T cells, both MIX and RNA showed increased levels of TM Tc17 CD69^+^, TM Tc0/Tc2, and TM Th0/Th2 PD1^+^ CXCR5^+^ if compared to those of REC subjects. Furthermore, RNA showed a higher percentage of TM Tc1 PD1^+^ CXCR5^+^ compared to those of REC and MIX (**Supplementary Figure S7**). Ag^+^CD8^+^ T cells were selected and after reclustering, 11 clusters were identified. Besides naive T cells and T_SCM_, defined as CD45RA^+^CCR7^+^CD27^+^CD28^+^CD95^−^ and CD45RA^+^CCR7^+^CD27^+^CD28^+^CD95^+^, respectively, we found two clusters of CM T cells defined as follows: CM Tc1 PD-1^−^ that were CD45RA^−^CCR7^+^CD28^+^CXCR3^+^PD-1^−^ and CM Tc1 PD-1^+^ that were CD45RA^−^CCR7^+^CD28^+^CXCR3^+^PD-1^+^.

Among effector Ag^+^CD8^+^ T cells, we found five clusters defined as TM Tc1 PD-1^−^ (CCR7^−^CD45RA^−^CD28^+^CXCR3^+^PD-1^−^), TM Tc1 PD-1^+^ (CCR7^−^CD45RA^−^CD28^+^CXCR3^+^PD-1^+^), TM Tc0/Tc2 (CCR7^−^CD45RA^−^CD28^+^CCR6^−^CXCR3^−^), TM Tc17 (CCR7^−^CD45RA^−^CD28^+^CCR6^+^CXCR3^−^), and EM Tc1 CD57^+^ PD-1^+^ (CCR7^−^CD45RA^−^CD28^−^CD57^+^PD-1^+^). Moreover, three populations of effector memory cells reexpressing CD45RA (EMRA) were detected, i.e., EMRA Tc1 CD57^−^ PD-1^+^ (CCR7^−^CD45RA^+^CXCR3^+^CD57^−^PD-1^+^), EMRA Tc1 CD57^+^ PD-1^+^ (CCR7^−^CD45RA^+^CXCR3^+^CD57^+^PD-1^+^), and EMRA Tc1 CD57^+^ PD-1^−^ (CCR7^−^CD45RA^+^CXCR3^+^CD57^+^PD-1^−^) (**Figure 2C**).

Similar percentages of total CD8^+^ and Ag^+^CD8^+^ T cells were found among the three groups (**Figures 2D, E**). However, within the Ag^+^ population, we observed increased percentages of EM Tc1 CD57^+^ PD-1^+^ in both vaccinated groups if compared to that in the recovered ones (**Figure 2F**). Furthermore, MIX and RNA showed increased levels of EMRA Tc1 CD57^+^ PD-1^+^ terminal effector CD8^+^ T cells compared to that in REC. Finally, we observed that the percentage of EMRA Tc1 CD57^+^ PD-1^−^ terminal effector CD8^+^ T cells was higher in MIX compared to those of both REC and RNA (**Figure 2E**). Similar percentages of all other subpopulations were found among REC, MIX, and RNA (**Supplementary Figure S8**).

### Patients who recovered from COVID-19 display more polyfunctional antigen-specific CD4^+^ T cells compared to those in vaccinated donors

Besides Th-polarization, the functional properties of Ag^+^-specific T cells were investigated by measuring the percentages of cells producing IFN-γ, tumor necrosis factor (TNF), interleukin (IL)-2, IL-17, and granzyme B (GZMB), along with the expression of the degranulation marker CD107a. The percentages of cells producing cytokines were assessed following 16 h of *in vitro* stimulation with SARS-CoV-2 peptide pool covering the complete sequence of Wuhan SARS-CoV-2 spike glycoprotein. The gating strategy is reported in **Supplementary Figure S9**.

**Figure 3:**
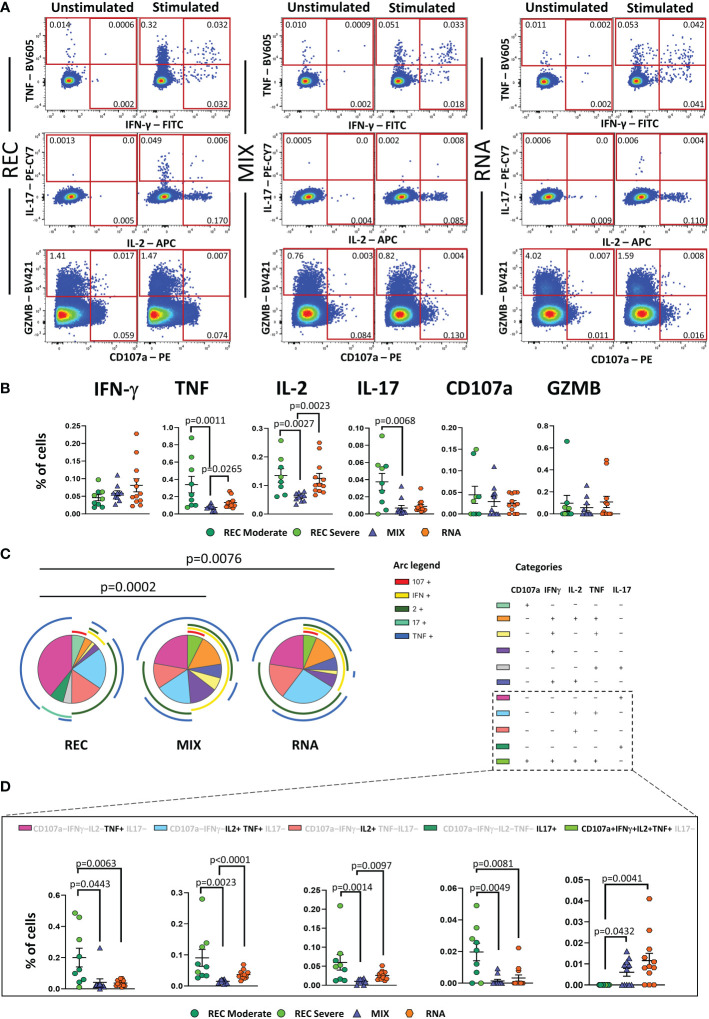
Cytokine production and polyfunctionality of antigen-specific CD4^+^ T cells. **(A)** Representative dot plots showing manual gating analysis of intracellular cytokine production of CD4^+^ T lymphocytes after overnight stimulation with spike protein compared to unstimulated control. Numbers in the dot plots indicate the percentage of cells identified by the gates. **(B)** Comparison between the total production of IFN-γ, TNF, IL-17, IL-2, CD107a, and GZMB by CD4^+^ T cells after *in vitro* stimulation with 15-mer peptides, covering the complete sequence of Wuhan SARS-CoV-2 spike glycoprotein. Data represent individual values from nine healthy subjects who recovered from SARS-CoV-2 infection (REC, severe = 4 and moderate = 5) and 23 vaccinated donors (MIX= 11 and RNA = 12). Mean (center bar) ± SEM (upper and lower bars). Kruskal–Wallis test with Benjamini–Hochberg correction for multiple comparisons was used to test the differences among the three groups. **(C)** Pie charts representing the proportion of responding CD4^+^ T cells producing different combinations of CD107a, IL-2, IL-17, IFN-γ, and TNF after *in vitro* stimulation with 15-mer peptides, covering the complete sequence of Wuhan SARS-CoV-2 spike glycoprotein. Frequencies were corrected by background subtraction as determined in non-stimulated controls using SPICE software. Pie arches represent the total production of different cytokines. **(D)** Percentage of polyfunctional population within CD4^+^ T cells. Kruskal–Wallis test with Benjamini–Hochberg correction for multiple comparisons was used to test the differences among the three groups. Adjusted P-values are indicated in the figure.

REC displayed a higher percentage of CD4^+^ T cells producing TNF, IL-2, and IL-17 than that in the MIX group, but not with respect to that of the RNA group. Furthermore, a higher percentage of CD4^+^ T cells producing IL-2 and TNF was observed in RNA compared to that in MIX subjects. Similar percentages of IFN-γ, CD107a, and GZMB were found among the three groups (**Figure 3A, B**).

Polyfunctional properties were investigated in CD4^+^ and CD8^+^ T cells by analyzing the simultaneous production of TNF, CD107a, IFN-γ, IL-2, and IL-17 using the bioinformatic Simplified Presentation of Incredibly Complex Evaluation (SPICE) tool. Among CD4^+^ T cells, REC exhibited a different polyfunctionality profile from those who had been vaccinated (**Figure 3C**). In particular, REC displayed a higher percentage of CD4^+^ T cells simultaneously producing IL-2 and TNF compared to those in MIX and RNA. The percentage of CD4^+^ T cells producing TNF or IL-17 was higher in REC compared to those in both vaccinated groups. Moreover, RNA exhibited higher percentages of CD4^+^ T cells simultaneously producing IL-2 and TNF or IL-2 alone compared to those in MIX. Furthermore, we found that both vaccinated groups displayed higher percentages of cells defined as “highly polyfunctional” as simultaneously producing CD107a, IFN-γ, IL-2, and TNF compared to those in REC (**Figure 3D**). The functional properties of CD8^+^ T were similar between the three groups (**Supplementary Figure S10**).

### Vaccinated donors showed a higher percentage of antigen-specific and activated memory B cells expressing IgG compared to that in REC

SARS-CoV-2 antibodies decline already as early as 21 days after infection or vaccination (6). However, long-lived MBCs constitute a durable long-term memory and provide a rapid recall response differentiating into high-affinity matured plasma cells (35). For this reason, we measured the frequencies of circulating SARS-CoV-2 spike-specific B cells (Ag^+^ B cells) (see *Methods*).

**Figure 4:**
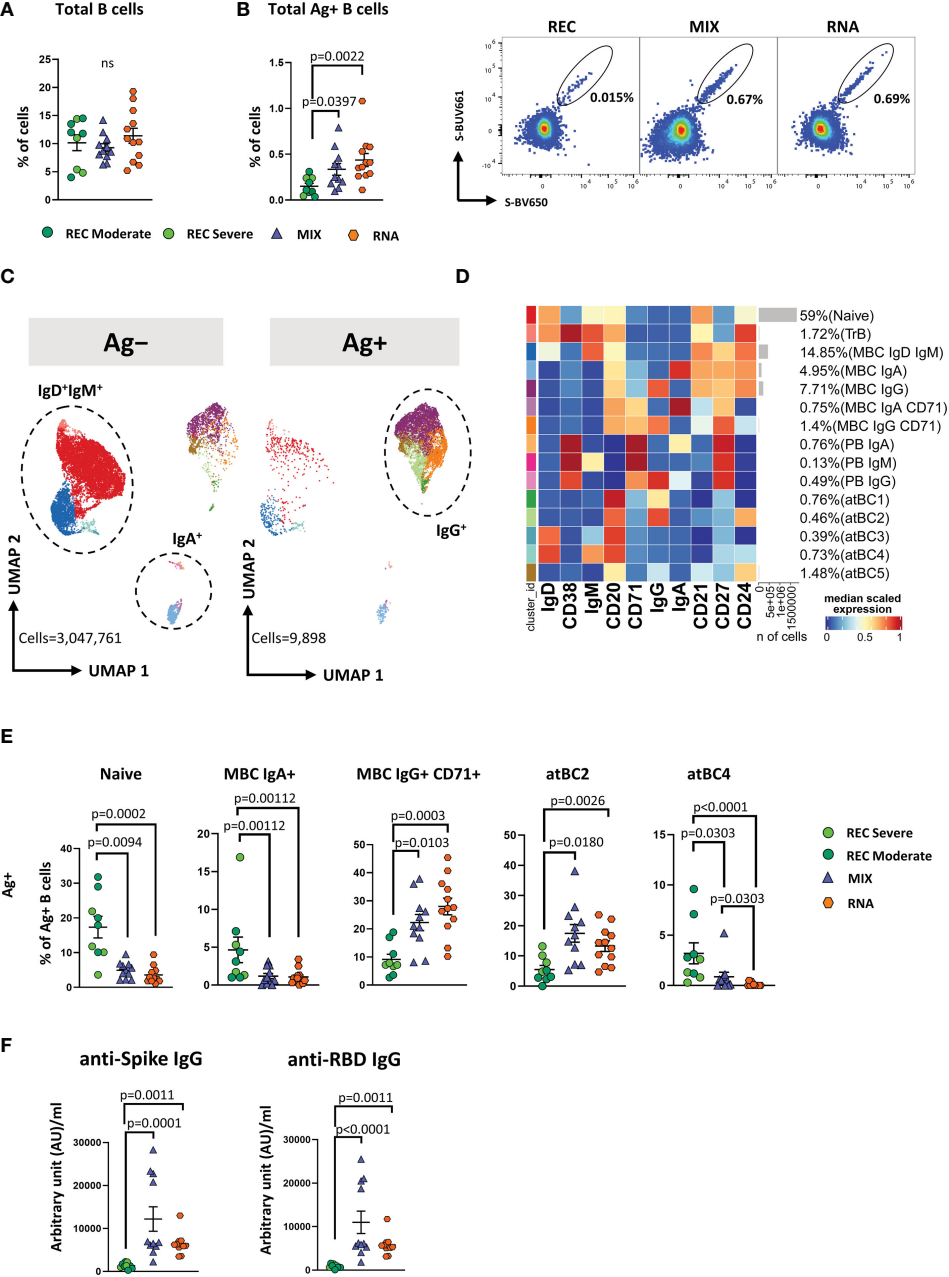
Immune phenotyping of antigen-specific CD19^+^ B cells. **(A)** Dot plots show the total percentage of CD19^+^ B cells. Kruskal–Wallis test with Benjamini–Hochberg correction for multiple comparisons was used to test the differences among the three groups. **(B)** Dot plots show the total percentage of antigen-specific CD19^+^ B cells (left); representative dot plots showing manual gating analysis of Ag^+^ B cells from REC, MIX, and RNA. Numbers in the dot plots indicate the percentage of cells identified by the gates (right). Kruskal–Wallis test with Benjamini–Hochberg correction for multiple comparisons was used to test the differences among the three groups. **(C)** UMAP plot shows the 2D spatial distribution of 3,057,659 cells from nine donors who recovered from SARS-CoV-2 infection (REC, severe = 4 and moderate = 5) and 23 vaccinated donors (MIX = 11 and RNA = 12) embedded with FlowSOM clusters. Ag^+^, antigen-specific CD19^+^ B cells; Ag^−^, non-antigen-specific CD19^+^ B cells. **(D)** Heatmap of the median marker intensities of the 10 lineage markers across the 15 cell populations obtained with FlowSOM algorithm after the manual metacluster merging. The colors of cluster_id column correspond to the colors used to label the UMAP plot clusters. The color in the heatmap is referred to the median of the *arcsinh* marker expression (0–1 scaled) calculated over cells from all of the samples. Blue represents lower expression, while red represents higher expression. Light gray bar along the rows (clusters) and values in brackets indicate the relative sizes of the clusters. N, naive; TrB, transitional B cells; MBC, memory B cell; atBC, atypical B cell. **(E)** Dot plots show the percentage of 15 Ag^+^ B cell clusters among nine donors who recovered from SARS-CoV-2 infection (REC, severe = 4 and moderate = 5) and 23 vaccinated donors (MIX = 11 and RNA = 12). The central bar represents the mean ± SEM. GLMM test was used for the statistical analysis. Adjusted P-values are reported in the figure. **(F)** Anti-spike and anti-RBD IgG concentrations in plasma samples from REC, MIX, and RNA individuals. Kruskal–Wallis test with Benjamini–Hochberg correction for multiple comparisons was used to test the differences among the three groups. Adjusted P-values are indicated in the figure.

Similar percentages of total B cells were found among the three groups (**Figure 4A**). However, both MIX and RNA showed a higher percentage of Ag^+^ B cells (defined as CD45^+^CD19^+^decoy^−^Spike-BUV661^+^Spike-BV650^+^) when compared to that in REC (**Figure 4B**).

By applying manual gating, we observed that Ag^+^ B cells compared to its Ag^−^ counterpart displayed a lower percentage of naive B cells and an increased percentage of memory switched, memory unswitched and of CD27^−^IgD^−^ B cells (**Supplementary Figure S11A**). Moreover, after vaccination or SARS-CoV-2 infection, ~42%–96% of Ag^+^ B cells were IgG^+^. This percentage decreased to ~5%–22% in the Ag^−^ B cells, where ~69%–90% of cells were IgD^+^IgM^+^ (**Supplementary Figure S11B**). Furthermore, the percentage of IgA^+^ B cells was higher in the Ag^−^ compartment (**Supplementary Figure S11B**).

To deeply characterize both Ag^−^ and Ag^+^ B cells, we took advantage of unsupervised clustering. The analysis revealed 15 clusters, spanning from naive to atypical B cells (atBCs; CD21^−^CD27^−^CD38^−^) (36) (**Figures 4C, D**; **Supplementary Figure S12A**).

Besides naive and transitional B cells (TrB), respectively defined as naive: CD20^+^CD21^+^CD24^+^CD38^−^IgD^+^IgM^+^ and TrB: CD20^+^CD21^+^CD24^+^CD38^+^IgD^+^IgM^+^, we found five clusters of MBCs defined as follows: MBC IgD^+^ IgM^+^ (CD20^+^CD21^+^CD24^+^CD27^+^IgD^+^IgM^+^), MBC IgA^+^ (CD20^+^CD21^+^CD24^+^CD27^+^IgA^+^), MBC IgG^+^ (CD20^+^CD21^+^CD24^+^CD27^+^IgG^+^), MBC IgA^+^ CD71^+^ (CD20^+^CD21^+^CD24^+^CD27^+^IgA^+^CD71^+^), and MBC IgG^+^ CD71^+^ (CD20^+^CD21^+^CD24^+^CD27^+^IGA^+^CD71^+^). Among plasmablasts (PBs), we found the following three clusters: PB IgA^+^ as CD27^+^CD71^+^CD38^++^IgA^+^, PB IgM^+^ as CD27^+^CD71^+^CD38^++^IgM^+^, and PB IgG^+^ as CD27^+^CD71^+^CD38^++^IgG^+^. Together with naive, TrBs, MBCs, and PBs, we identified five clusters of atBCs, i.e., atBC1 as CD21^−^CD27^−^CD20^+^IgG^+^, atBC2 as CD21^−^CD27^−^CD24^+^CD20^+^IgG^+^, atBC3 as CD21^−^CD27^−^CD20^+^IgD^+^, atBC4 as CD21^−^CD27^−^CD20^+^IgD^+^IgM^+^, and atBC5 as CD21^−^CD27^−^CD20^+^CD24^+^.

Within Ag^−^ B cells, MIX and RNA showed higher levels of MBC IgD^+^IgM^+^ and lower levels of atBC5 compared to those in REC (**Supplementary Figure S13**). Within Ag^+^ B cells, MIX and RNA displayed lower percentages of naive, MBC IgA^+^, and atBC4 B cells if compared to those in REC, while the percentages of MBC IgG^+^ CD71^+^ and atBC2 were significantly higher (**Figure 4E**). Moreover, REC displayed a higher percentage of atBC4 cells if compared to those in MIX and RNA (**Figure 4E**). Similar percentages of all other subpopulations were found among REC, MIX, and RNA subjects (**Supplementary Figure S14**).

In addition, we measured IgG antibodies able to bind the spike and the RBD of the S1 subunit of the spike protein (the latter known as neutralizing antibodies). We observed that both vaccinated groups had higher levels of anti-spike and anti-RBD-binding IgG compared to those in REC subjects (**Figure 4F**).

### Recovered patients show different immunological profiles compared to those of vaccinated donors

**Figure 5:**
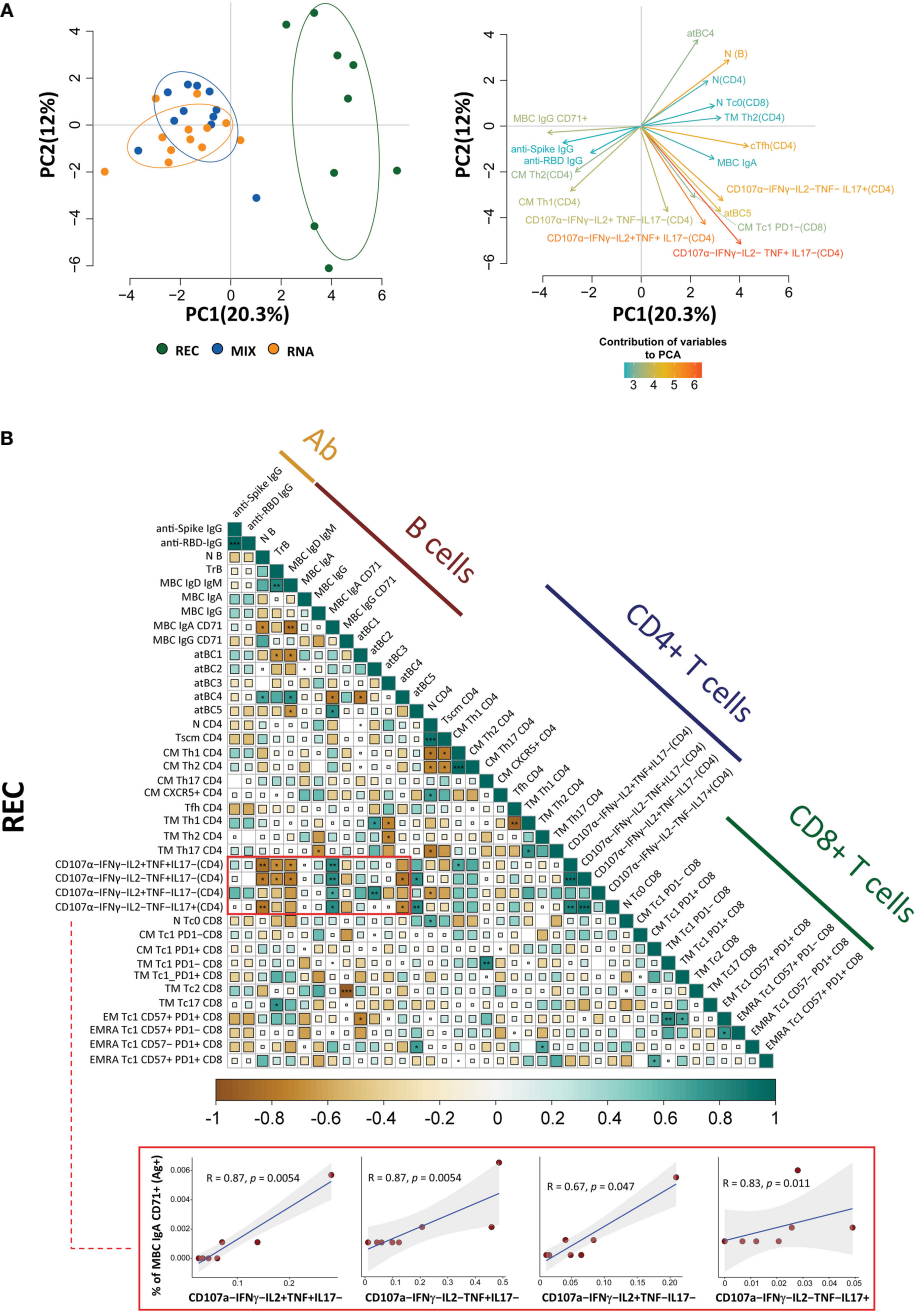
Principal component analysis and correlogram reveal that REC is different from MIX and RNA. **(A)** (Left) Principal component analysis (PCA) using the plasma level of anti-SARS-CoV-2 IgG antibodies, Ag-specific T, B-cell percentages, and the fraction of polyfunctional CD4^+^ T cells from REC, MIX, and RNA subjects. REC, green circles (n = 9); MIX, blue circles (n = 11); RNA, orange circles (n = 12). (Right) Contribution of the different variables to PCA. The color of the arrows underlines the contribution level, while the position underlines the positive or negative contribution. Negatively correlated variables are positioned on opposite sides of the plot origin (opposed quadrants). **(B)** Correlogram of REC. Spearman R (ρ) values are shown from brown (−1.0) to green (1.0); color intensity and areas of square are proportional to correlation coefficients R. Spearman rank two-tailed P-value was indicated by *P < 0.05, **P < 0.01, and ***P < 0.001. Additional XY scatter plots that specifically show the relationship between the variables that are most correlated are displayed. Each scatter plot reports the regression line (blue), the Spearman R (ρ) value, the exact two-tailed P-value, and the 95% confidence bands (light gray).

The principal component analysis (PCA) computed using the complete phenotype of Ag^+^ B and T cells, CD4^+^ T cell polyfunctionality, plasmatic anti-spike, and anti-RBD antibodies showed that the group of REC clusters in a different position of the two-dimensional PCA space if compared to MIX and RNA, which are almost entirely overlapping (**Figure 5A**, left). Immune features related to the amount of MBC IgA, CD107a^−^IFN-γ^−^IL2^−^TNF^+^IL17^−^, CD107a^−^IFN-γ^−^IL2^+^TNF^+^IL17^−^, CD107a^−^IFN-γ^−^IL2^−^TNF^−^IL17^+^, and naive B cells (more abundant in REC subjects) were the main drivers of the clusterization of samples in two different areas (**Figure 5A**, right). Moreover, the picture of PCA contribution also reveals that both vaccinated groups were characterized by increased levels of MBC IgG CD71^+^, anti-spike, and anti-RBD IgG antibodies (**Figure 5A**, right).

By using the same parameters used to perform the PCA, we assessed the existence of immunological correlations between the variables within the REC, MIX, and RNA groups. It is to note that in REC, but not in the MIX and RNA groups, a strong positive correlation was present among the percentages of MBC IgA CD71^+^ and all polyfunctional CD4^+^ T-cell subsets (**Figure 5B**, **Supplementary Figure S15**). The percentages of MBC IgD^+^ IgM^+^, transitional, and naive B cells inversely correlate with all polyfunctional CD4^+^ T-cell subsets (**Figure 5B**, **Supplementary Figure S15**).

## Discussion

Vaccines are designed to induce a long-term adaptive immune response that confers durable protection. In this study, as revealed by PCA, we report that COVID-19 recovered patients show different long-term immunological profiles compared to those of donors who had been vaccinated with three doses (either with adenovirus or mRNA technologies). Vaccinated individuals display a skewed Th1 Ag-specific T-cell polarization and a higher percentage of Ag-specific and activated MBCs expressing IgG compared to those of patients who recovered from severe COVID-19. Different polyfunctional properties characterize the two groups: recovered individuals show higher percentages of CD4^+^ T cells producing one or two cytokines simultaneously, while vaccinated donors are distinguished by highly polyfunctional populations able to release four molecules such as CD107a, IFN-γ, TNF, and IL-2.

SARS-CoV-2 entry route shapes the innate immune response, as major players such as macrophages and neutrophils contribute to recruit T and B cells that should mount a local specific immune response, with the consequent production of mucosal antibodies. This means that different adaptive mechanisms are involved in the protective immunity generated by the infection or vaccination. Indeed, we found that recovered individuals are characterized by higher percentages of MBCs producing IgA if compared to those of vaccinated ones. However, systemic and mucosal IgA responses are variably induced in response to vaccination and are associated with protection against subsequent infection (37, 38).

SARS-CoV-2-specific cells wane more slowly than do antibodies [reviewed in (39)], and T cells able to exert an efficient protection are those capable of exerting many functions simultaneously. Polyfunctional T-cell responses have been documented also in HIV-1 (40), hepatitis B virus vaccine (41), and vaccinia-induced responses (42), indicating that highly functional T-cell responses are commonly found in response to other viral infections and vaccination and are effectively controlled by cellular immunity. The functional population able to produce four cytokines or more is likely of significant immunologic importance because it could directly eliminate virally infected cells (assuming that such cells express or upregulate CD107a) and suppress viral replication while maintaining itself without CD4^+^ T-cell help through autocrine production of IL-2.

We found that vaccinated donors are characterized by MBCs IgG-switched that express CD71. Ag-specific B cells can be divided into antibody-secreting cells (or PBs) and MBCs after infection or vaccination. A particular subset of B cells, called activated B cells, is distinct from antibody-secreting cells and is committed to the MBC lineage. Activated B cells are characterized by the expression of CD71, which is the transferrin receptor and indicates higher activation status and proliferative capabilities (43). This population is also typically found in blood after infection with Ebola or influenza virus and also after vaccination (44–46).

As far as vaccination strategy is concerned, the ChAdOx1 vaccine uses a nonreplicating adenovirus as a vector to introduce into the cells of the recipient’s DNA coding for the spike protein of SARS-CoV-2. BNT162b2 instead uses messenger RNA (mRNA) coding for spike, which cells take up and use to synthetize the protein. mRNA vaccines are good at inducing antibody responses, and the vector-based vaccines are better at triggering T-cell responses. In a Spanish study, people who received a dose of the Pfizer-BioNTech vaccine 8 weeks after an initial AstraZeneca dose had few side effects and a robust antibody response 2 weeks after the second shot, suggesting that mixing the two types of vaccine may give the immune system multiple ways to recognize a pathogen (47). However, in our small cohort of vaccinated individuals, the immunological response was not different in the two groups of individuals who received different vaccination strategies likely because the immune response has been investigated after the third dose.

We are well aware that this study has some limitations. First of all, the number of patients studied is low, but the B- and T-cell compartments were investigated in-depth in terms of phenotype and functionality. Second, the number of days post symptom onset (for recovered individuals) or after the third dose of the vaccine (for vaccinated ones) is different. This could be relevant when interpreting the results regarding SARS-CoV-2-specific antibodies or the percentage of Ag-specific B cells and cTfh cells in recovered patients. Third, a group of donors who developed hybrid immunity characterized by immunity developed by natural infection and vaccination.

However, our study can provide a novel characterization of the humoral and cellular immune responses upon COVID-19 vaccination or infection by including the fine phenotypic and functional analysis of Ag-specific B and T cells together with the comparison between different vaccination strategies (after the third dose of vaccination) and natural infection.

## Methods

### Blood collection and isolation of mononuclear cells

Up to 30 ml of blood was collected from each patient in vacuettes containing ethylenediaminetetraacetic acid (EDTA). Blood was immediately processed. Isolation of PBMCs was performed using Ficoll-Hypaque according to standard procedures (48). PBMCs were stored in liquid nitrogen in fetal bovine serum (FBS) supplemented with 10% dimethyl sulfoxide (DMSO). Plasma was stored at -80°C until use.

### Activation-induced cell marker assay and T-cell phenotype

Isolated PBMCs were thawed and rested for 6 h. After resting, CD40-blocking antibody (0.5 µg/ml final concentration) (Miltenyi Biotec, Bergisch Gladbach, Germany) was added to the cultures 15 min before stimulation. PBMCs were cultured in a 96-well plate in the presence of 15-mer peptides with 11-amino acid overlap, covering the complete sequence of Wuhan SARS-CoV-2 spike glycoprotein (PepTivator SARS-CoV-2 Prot_S complete, Miltenyi Biotec, Bergisch Gladbach, Germany) together with 1 μg/ml of anti-CD28 (Miltenyi Biotec, Germany). PBMCs were stimulated for 18 h at 37°C in a 5% CO_2_ atmosphere in complete culture medium (RPMI 1640 supplemented with 10% FBS and 1% each of L-glutamine, sodium pyruvate, nonessential amino acids, antibiotics, 0.1 M 4-(2-hydroxyethyl)-1-piperazineethanesulfonic acid (HEPES), and 55 μM β-mercaptoethanol) (33, 34). For each stimulated sample, an unstimulated one was prepared as a negative control. After stimulation, cells were washed with Phosphate buffer saline (PBS) and stained with PromoFluor IR-840 (Promokine, PromoCell, Heidelberg, Germany) for 20 min at room temperature (RT). Next, cells were washed with FACS buffer (PBS added with 2% FBS) and stained with the following fluorochrome-labeled monoclonal antibodies (mAbs) for 30 min at 37°C: CXCR5-BUV661, CCR6-BUV496, and CXCR3-BV785. Finally, cells were washed with FACS buffer and stained for 20 min at RT with Duraclone IM T-cell panel (Beckman Coulter, Brea, CA, USA) containing CD45-Krome Orange, CD3-APC-A750, CD4-APC, CD8-AF700, CD27-PC7, CD57-Pacific Blue, CD279 (PD1)-PC5.5, CD28-ECD, CCR7-PE, and CD45RA-FITC and added with three other fluorescent mAbs, i.e., CD69-BV650, CD137-BUV395, and CD95-BV605. Samples were acquired on a CytoFLEX LX flow cytometer (Beckman Coulter). All reagents used for T-cell phenotype are reported in **Supplementary Table S1**. All mAbs added to DuraClone IM T cells were previously titrated on human PBMCs and used at the concentration giving the best signal-to-noise ratio. The gating strategy used to identify CD4^+^ and CD8^+^ T cells is reported in **Supplementary Figure S16**.

### Intracellular cytokine staining

Isolated PBMCs were thawed and rested for 6 h. PBMCs were then cultured in the presence of 15-mer peptides with 11-amino acid overlap, covering the complete sequence of Wuhan SARS-CoV-2 spike glycoprotein (PepTivator SARS-CoV-2 Prot_S complete, Miltenyi Biotec, Bergisch Gladbach, Germany) together with 1 μg/ml of anti-CD28 (Miltenyi Biotec, Germany). PBMCs were stimulated for 16 h at 37°C in a 5% CO_2_ atmosphere in complete culture medium (RPMI 1640 supplemented with 10% FBS and 1% each of L-glutamine, sodium pyruvate, nonessential amino acids, antibiotics, 0.1 M HEPES, and 55 μM β-mercaptoethanol). For each stimulated sample, an unstimulated one was prepared as a negative control. All samples were incubated with protein transport inhibitor containing brefeldin A (Golgi Plug, Becton Dickinson) and monensin (Golgi Stop, Becton Dickinson) and previously titrated concentration of CD107a-PE (BioLegend, San Diego, CA, USA). After stimulation, cells were washed with PBS and stained with LIVE/DEAD fixable Aqua (ThermoFisher Scientific, USA) for 20 min at RT. Next, cells were washed with FACS buffer and stained with surface mAbs recognizing CD3-PE.Cy5, CD4-AF700, and CD8-APC.Cy7 (BioLegend, San Diego, CA, USA). Cells were washed with FACS buffer and fixed and permeabilized with the Cytofix/Cytoperm buffer set (Becton Dickinson Bioscience, San Jose, CA, USA) for cytokine detection. Then, cells were stained with previously titrated mAbs recognizing IL-17-PE-Cy7, TNF-BV605, IFN-γ-FITC, IL-2-APC, and GZMB BV421 (all mAbs from BioLegend). Samples were acquired on an Attune NxT acoustic cytometer (ThermoFisher Scientific, USA). **Supplementary Table S1** reports mAb titers, clones, catalog numbers, and type of fluorochrome used in the panel. Gating strategy used to identify and analyze the intracellular cytokine production of CD4^+^ and CD8^+^ T lymphocytes is reported in **Supplementary Figure S9**.

### Detection of SARS-CoV-2-specific B cells

Thawed PBMCs were washed twice with RPMI 1640 supplemented with 10% FBS and 1% each of L-glutamine, sodium pyruvate, nonessential amino acids, antibiotics, 0.1 M HEPES, 55 μM β-mercaptoethanol, and 0.02 mg/ml DNAse. PBMCs were washed with PBS and stained using viability marker PromoFluor IR-840 (Promokine, PromoCell, Heidelberg, Germany) for 20 min at RT in PBS. Next, cells were washed with PBS and stained for 15 min at RT with streptavidin-AF700 (decoy channel; ThermoFisher Scientific, USA) to remove false-positive SARS-CoV-2-specific B cells. After washing with FACS buffer, cells were stained with biotinylated full-length SARS-CoV-2 spike protein (R&D Systems, Minneapolis, USA) labeled with different streptavidin-fluorophore conjugates. Full-length biotinylated spike protein was mixed and incubated with streptavidin-BUV661 (Becton Dickinson) or streptavidin-BV650 (BioLegend) at a 6:1 mass ratio for 15 min at RT. All samples were stained with both fluorescent biotinylated biotinylated spike protein for 1 h at 4°C. Then, cells were washed with FACS buffer and stained for 20 min at RT with DuraClone IM B cells (Beckman Coulter, Brea, CA, USA) containing the following lyophilized directly conjugated mAbs: anti-IgD-FITC, CD21-PE, CD19-ECD, CD27-PC7, CD24-APC, CD38-AF750, anti-IgM-PB, and CD45-KrO to which the following drop-in antibodies were added: CD71-BUV395, CD20-BV785, anti-IgG-BUV496, and anti-IgA-PerCP-Vio700. Samples were acquired on a CytoFLEX LX flow cytometer (Beckman Coulter). A minimum of 1,000,000 cells per sample were acquired. All reagents used for B-cell phenotype are reported in **Supplementary Table S1**. All mAbs added to DuraClone IM B cells were previously titrated on human PBMCs and used at the concentration giving the best signal-to-noise ratio. The gating strategy used to identify Ag^−^ and Ag^+^ B cells is reported in **Supplementary Figure S17**.

### Computational analysis of flow cytometry data

#### T-cell analysis

Compensated Flow Cytometry Standard (FCS) 3.0 files were imported into FlowJo software version v10.7.1 and analyzed by standard gating to remove doublets, aggregates, and dead cells. For *ex vivo* immunophenotyping of non-antigen-specific (Ag^−^) and antigen-specific (Ag^+^) T cells of both CD4^+^ and CD8^+^, we analyzed only the data of stimulated samples. For each sample, we therefore selected data from all living CD4^+^ or CD8^+^ T cells and imported them in R using flowCore package v2.4.0 (49) for a total of 8,436,275 CD4^+^ T cells (of which 89,400 were SARS-CoV-2-specific) and 3,723,899 CD8^+^ T cells (of which 20,413 were SARS-CoV-2-specific). Further analysis was performed using CATALYST v1.17.3 (50). All data obtained by flow cytometry were transformed in R using hyperbolic arcsine “*arcsinh* (x/cofactor)” applying manually defined cofactors (where x is the fluorescence-measured intensity value). Clustering and dimensional reduction were performed using FlowSOM (version 2.4.0) and Uniform Manifold Approximation and Projection (UMAP) (version 0.2.8.0) algorithms, respectively. The Ag^+^ CD4^+^ and CD8^+^ T-cell clusters have been reanalyzed more in-depth by performing a new step of clustering using the following markers: CD45RA, CCR7, CD27, CD28, PD-1, CCR6, CXCR3, CXCR5, and CD95. Starting from 15 clusters of either CD4^+^ T cells or CD8^+^ T cells, reclustering gave origin to 10 clusters of CD4^+^ T cells and 11 of CD8^+^ T lymphocytes. The quality control (QC) of clustering for CD4^+^ and CD8^+^ T cells is reported in **Supplementary Figures S2** and **S6**, respectively.

#### B-cell analysis

Compensated FCS 3.0 files were imported into FlowJo software version v10.7.1 and analyzed by standard gating to remove doublets, aggregates, and dead cells and identify CD19^+^ B cells. Then, from the total CD19^+^ B cells, we excluded decoy-positive B cells to remove false-positive SARS-CoV-2-specific B cells. For each sample, we selected the SARS-CoV-2-specific B cells as positive cells for both Spike_streptavidin-BUV661 and Spike_streptavidin-BV650 (we referred to as Ag^+^ B cells). The remaining double-negative cells were non-SARS-CoV-2-specific B cells (we referred to as Ag^−^ B cells). Then, we exported for each sample separately both Ag^+^ and Ag^−^ B cells and imported them in R using flowCore package v2.4.0 for a total of 3,057,659 CD19^+^ B cells (of which 9,898 were SARS-CoV-2-specific). The unsupervised analysis was performed using CATALYST v1.17.3. All data were transformed in R using hyperbolic arcsin (*arcsinh* x/cofactor) applying manually defined cofactors (where x is the fluorescence-measured intensity value). Clustering and dimensional reduction were performed using FlowSOM and UMAP algorithms, respectively. The QC of clustering for B cells is reported in **Supplementary Figure S12**.

#### Measuring anti-SARS-CoV-2 anti-spike and anti-RBD IgG antibodies

Anti-spike antibody levels were measured by qualitative and semiquantitative chemiluminescent microparticle immunoassay (CMIA). AdviseDx SARS-CoV-2 IgG II assay (Abbott) was used to detect plasmatic IgG antibodies able to bind the RBD of the S1 subunit of the spike protein. Plasma, SARS-CoV-2 Ag-coated paramagnetic microparticles, and assay diluent are combined and incubated. The anti-spike IgG antibodies present in the sample bind to the SARS-CoV-2 Ag-coated paramagnetic microparticles. The mixture was then washed. Anti-human IgG acridinium-labeled conjugate was added and incubated to create a reaction mixture. The resulting chemiluminescent reaction was measured as a relative light unit (RLU). There is a direct relationship between the amount of IgG antibodies to SARS-CoV-2 in the sample and the RLU detected by the system optics. Results from the anti-spike AdviseDx SARS-CoV-2 IgG II assay are reported as arbitrary units per milliliter (AU/ml). As recommended, we applied a cutoff of 50 AU/ml as a positive threshold. Every measurement was performed on Abbott “Alinity I” platform. The level of anti-RBD IgG antibodies was calculated by using NAB Neutralizing Antibody kit (SGM Italia).

#### Principal component analysis and correlation plot

PCA was performed and visualized in R using prcomp and pca3d package. To perform PCA, we used a matrix containing the level of plasmatic anti-SARS-CoV-2 IgG antibodies, Ag-specific T, B-cell percentages, and the fraction of polyfunctional CD4^+^ T cells (**Supplementary Table S2**). The total contribution of a given variable retained by PC1 and PC2 is equal to [(C1 * Eig1) + (C2 * Eig2)]/(Eig1 + Eig2), where C1 and C2 are the contributions of the variable on PC1 and PC2; Eig1 and Eig2 are the eigenvalues of PC1 and PC2.

Correlation analysis was performed on the same parameters used to run the PCA (see above) except the following features that were not used for the correlation analysis of REC donors because they were not available: PB IgA, PB, and CD107a^+^IFN-γ^+^IL2^+^TNF^+^IL17^−^. Pairwise correlations between variables were calculated and visualized as a correlogram using R packages stats (version 3.6.2) and corrplot (version 0.90). Spearman’s rank correlation coefficient (ρ) was indicated by color scale; significance was indicated by asterisks (* P < 0.05; ** P < 0.005; *** P < 0.0005).

#### Statistical analysis

Differential cell population abundance analysis was performed using generalized linear mixed model (GLMM) implemented within diffcyt package (51) applying FDR cutoff = 0.05; each P-value was reported in the figure. Quantitative variables were compared using Kruskal–Wallis nonparametric test corrected for multiple comparisons by controlling the false discovery rate (FDR), method of Benjamini and Hochberg. Statistically significant adjusted P-values are represented. Statistical analysis of cytokine production was performed using GraphPad Prism version 8 (GraphPad Software Inc., La Jolla, USA). The total percentage of Ag-specific (Ag^+^CD4^+^ and Ag^+^CD8^+^) T-cell data has been calculated as background subtracted data. SPICE software (version 6, kindly provided by Dr. Mario Roederer, Vaccine Research Center, NIAID, NIH, Bethesda, MD, USA) was used to analyze flow cytometry data on T-cell polyfunctionality (52). Data from the total cytokine production are represented as individual values, means, and standard errors of the mean. Regarding polyfunctionality, data in pie charts are represented as median values, and statistical analysis was performed using the permutation test. Data in graphs are represented as individual values, means, and standard errors of the mean.

## Data availability statement

The original contributions presented in the study are included in the article/**Supplementary Material**. Further inquiries can be directed to the corresponding author.

## Ethics statement

The studies involving human participants were reviewed and approved by Each participant, including healthy donors, provided informed consent according to Helsinki Declaration, and all uses of human material have been approved by the local Ethical Committee (Comitato Etico dell’Area Vasta Emilia Nord, protocol number 177/2020, March 11th, 2020) and by the University Hospital Committee (Direzione Sanitaria dell’Azienda Ospedaliero Universitaria di Modena, protocol number 7531, March 11th, 2020). The patients/participants provided their written informed consent to participate in this study.

## Author contributions

DT, AP, AN, RB, JS, LGi, ES, AD, MC carried out experiments. DT and AP drafted the figures. DT and AN drafted and revised the tables. MMe, MMa, MG, GG, SB, CM, TT followed patients. LGo drafted and revised the clinical tables. AP performed ICS analysis. DT and KP performed bioinformatic and statistical analyses. DT, SB and AC conceived the study. SB and AC wrote the manuscript. All authors contributed to the article and approved the submitted version.
