# Supplementary figures and images for: Detailed characterization of SARS-CoV-2-specific T and B cells after infection or heterologous vaccination
Source: Front Immunol. 2023 Feb 9;14:1123724. doi: 10.3389/fimmu.2023.1123724 (PMC9947839; doi:10.3389/fimmu.2023.1123724)

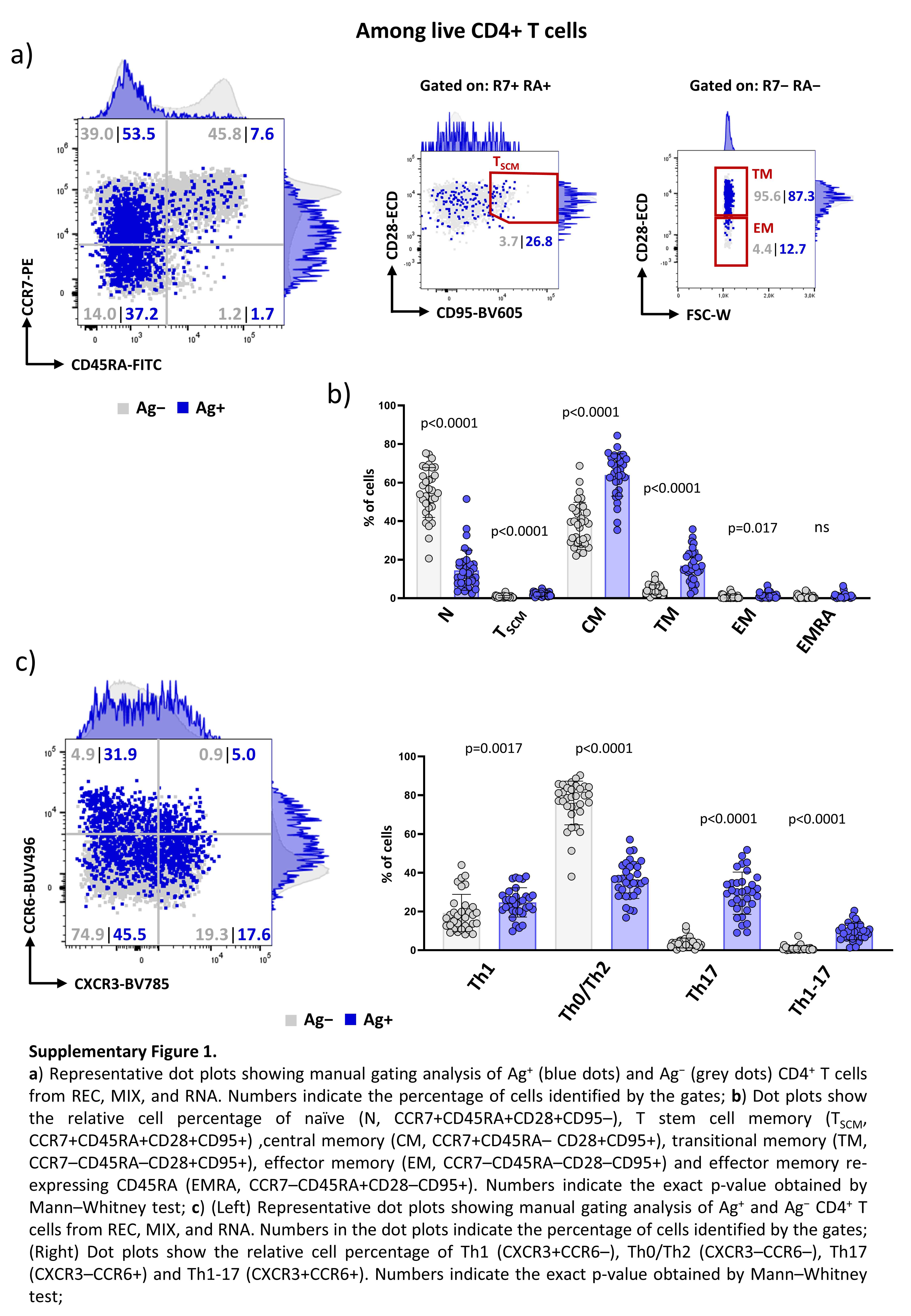

Supplement: Supplementary file 1 [file Image_1.tif]

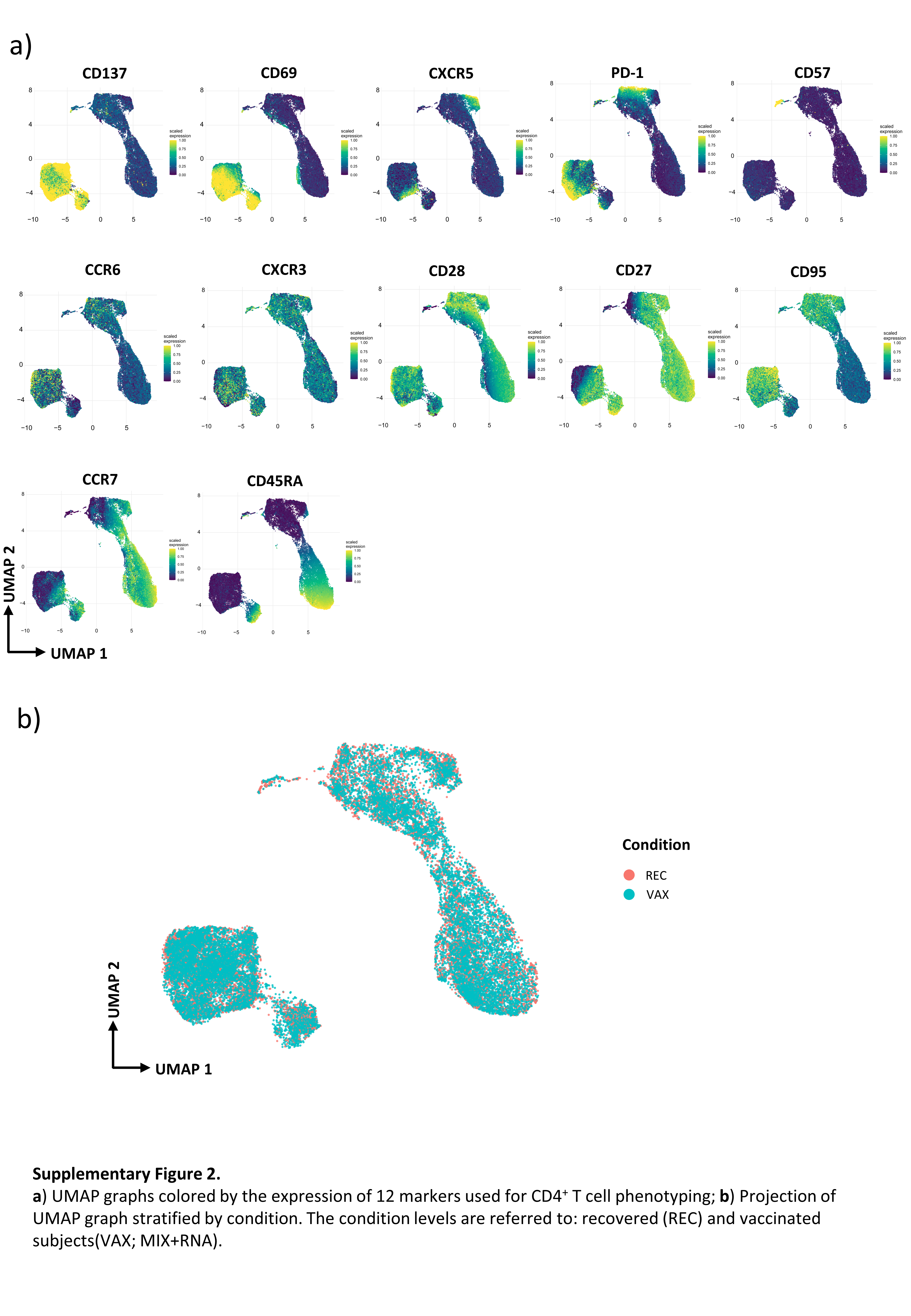

Supplement: Supplementary file 2 [file Image_2.tif]

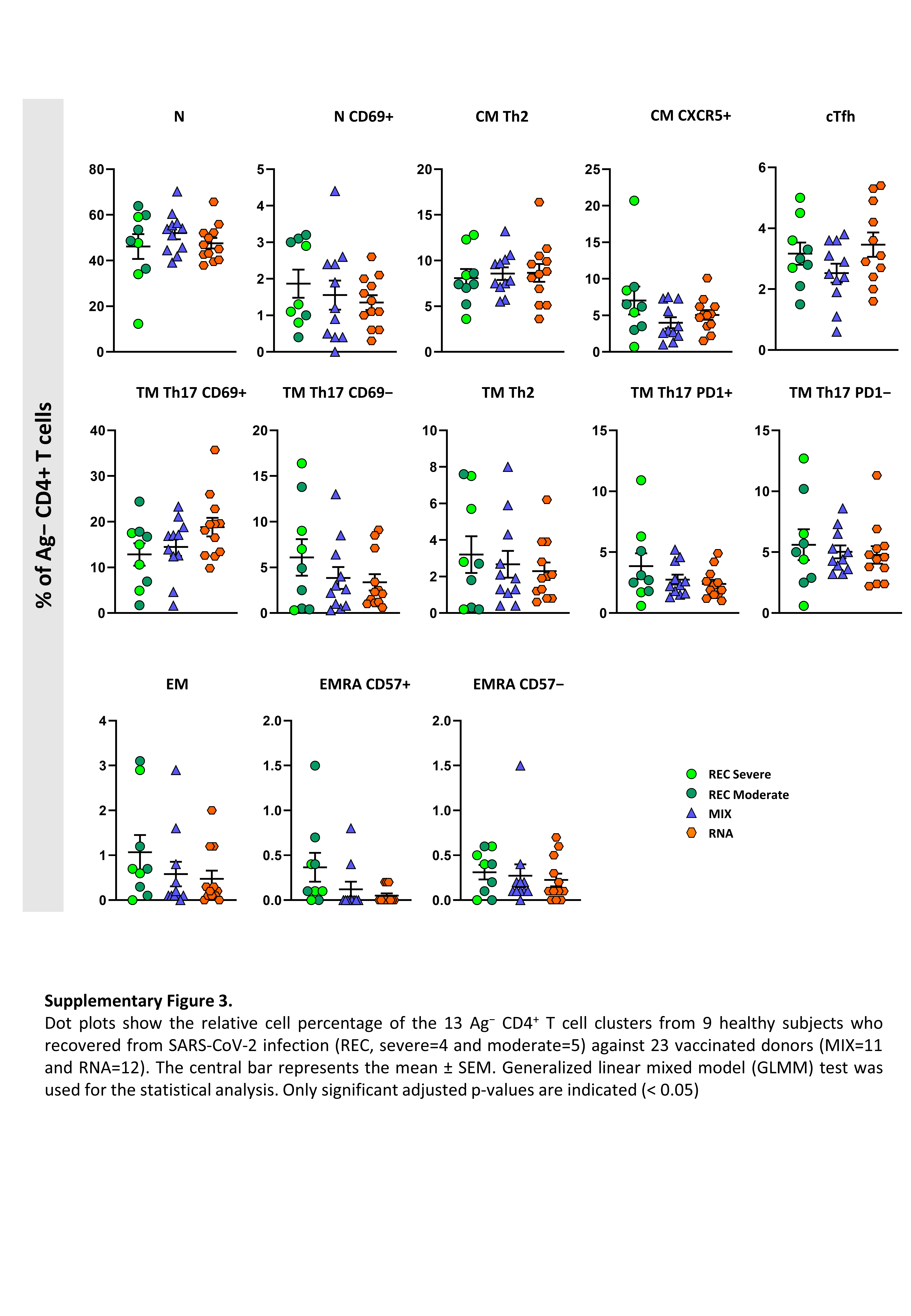

Supplement: Supplementary file 3 [file Image_3.tif]

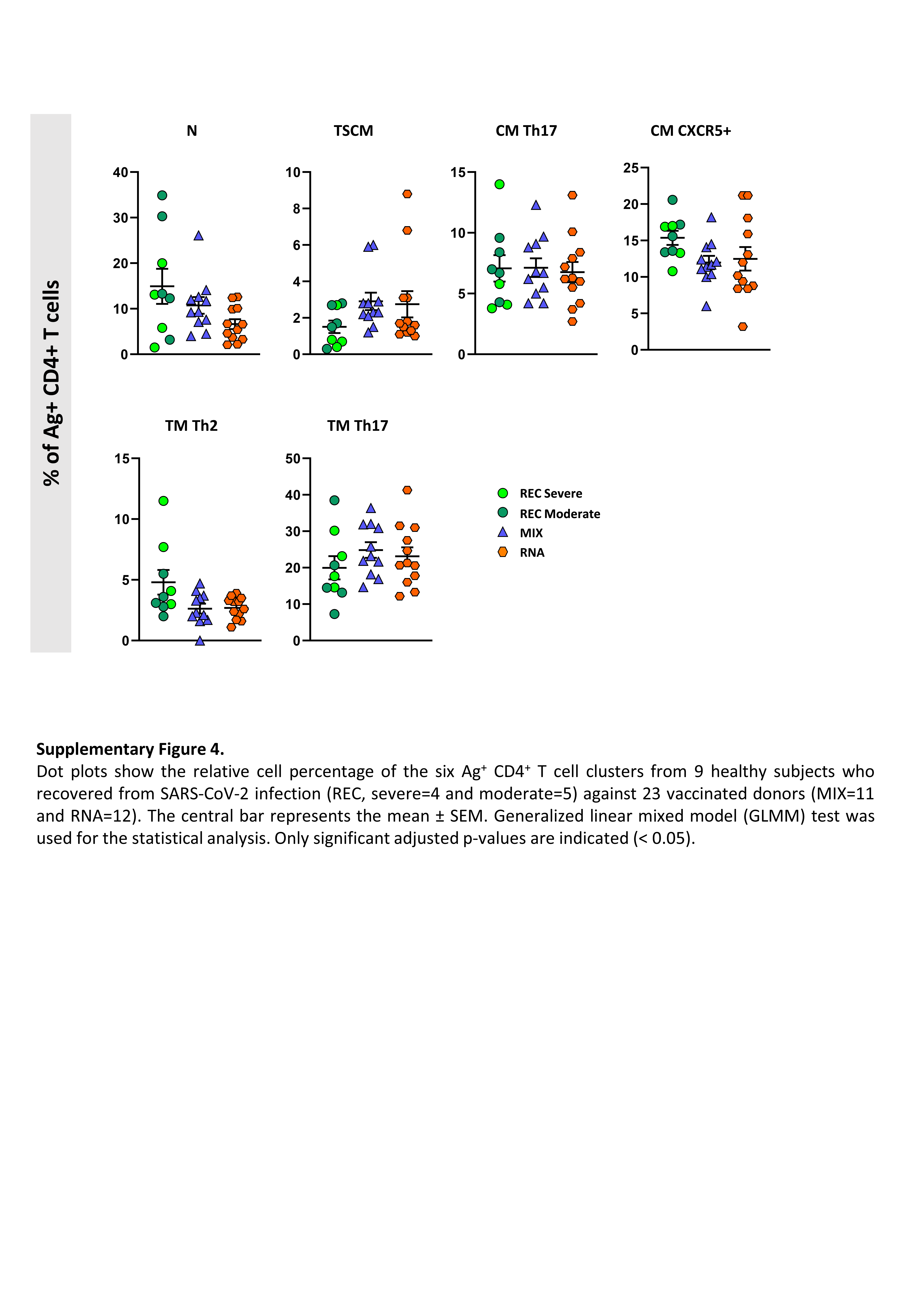

Supplement: Supplementary file 4 [file Image_4.tif]

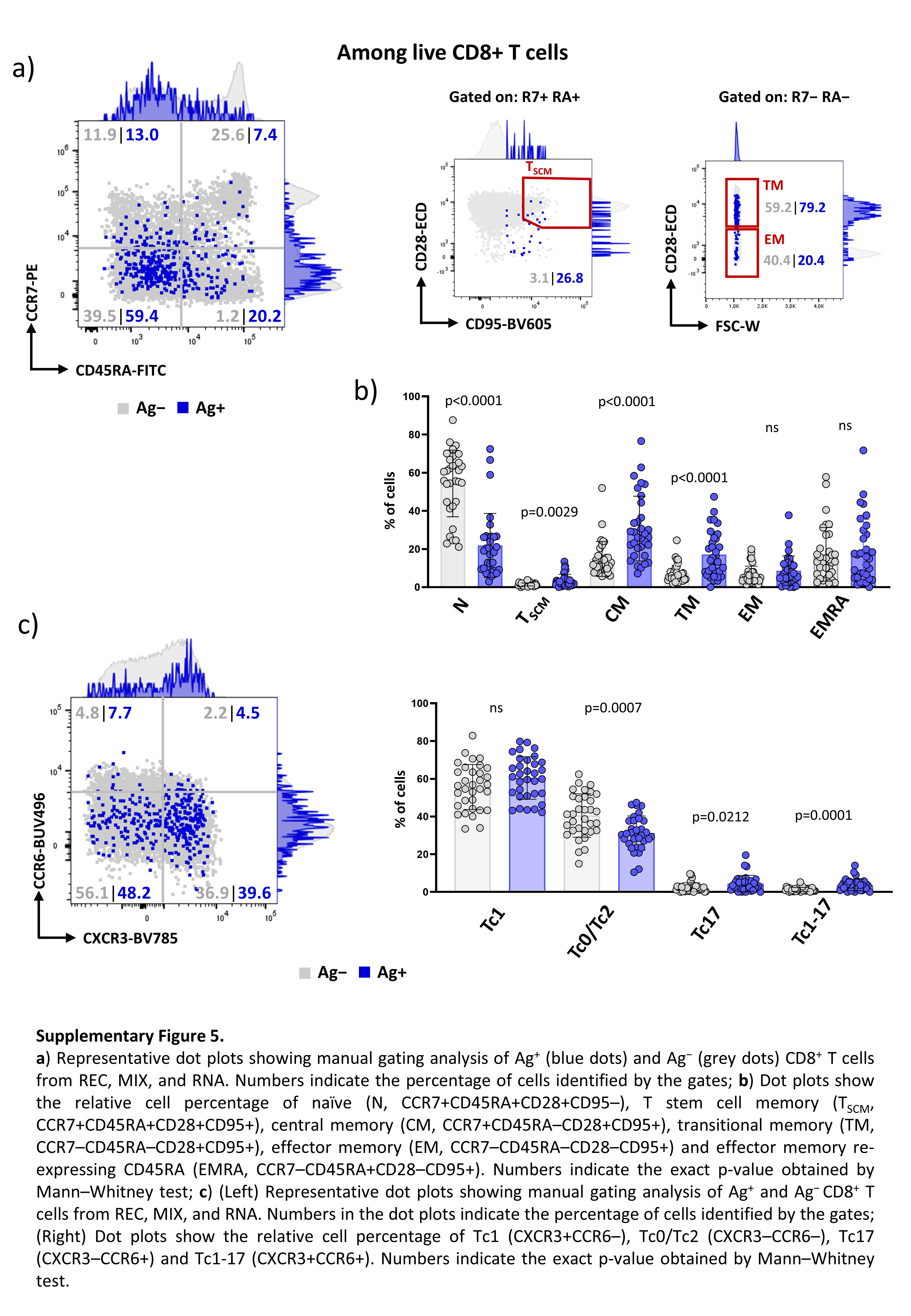

Supplement: Supplementary file 5 [file Image_5.tif]

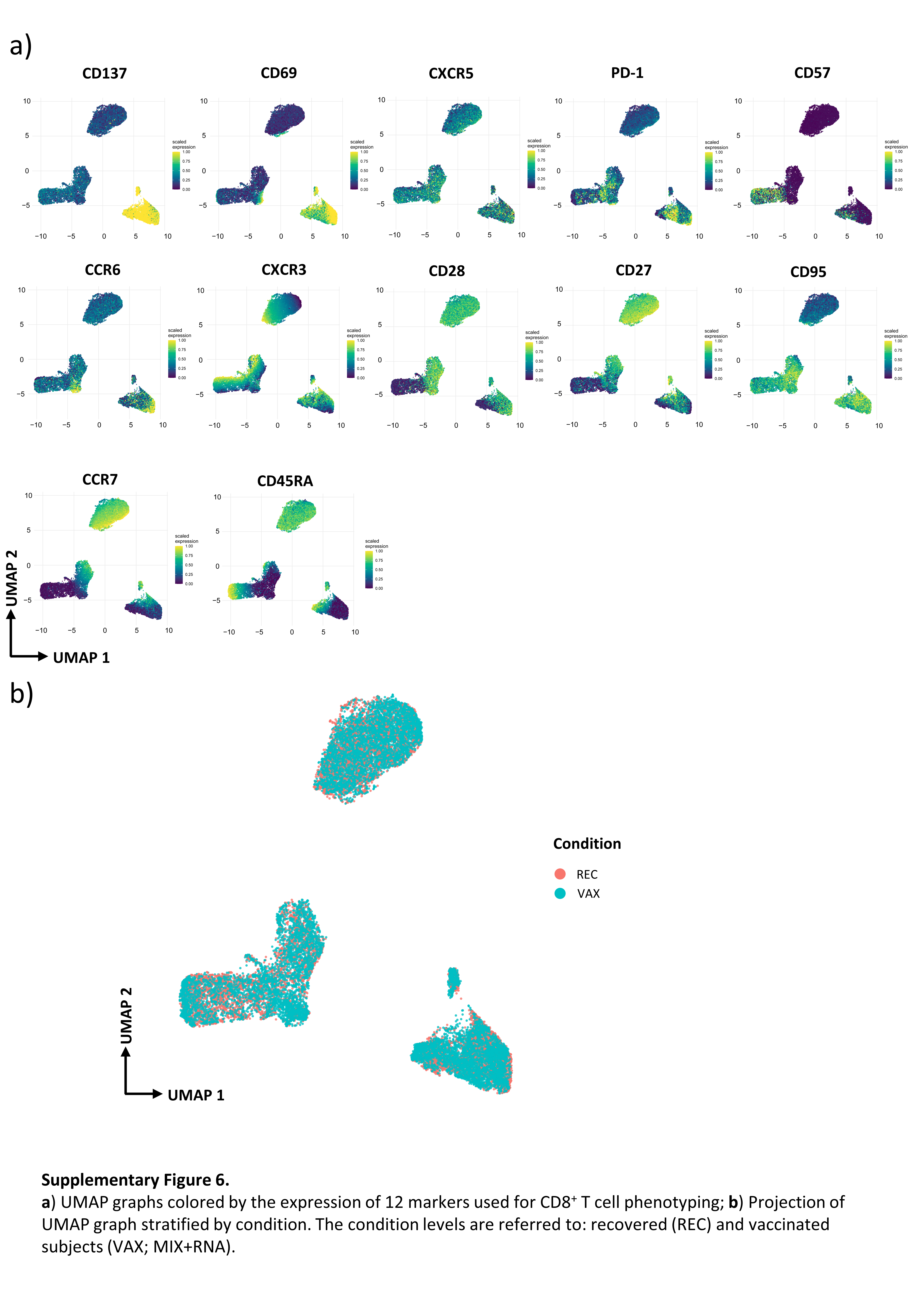

Supplement: Supplementary file 6 [file Image_6.tif]

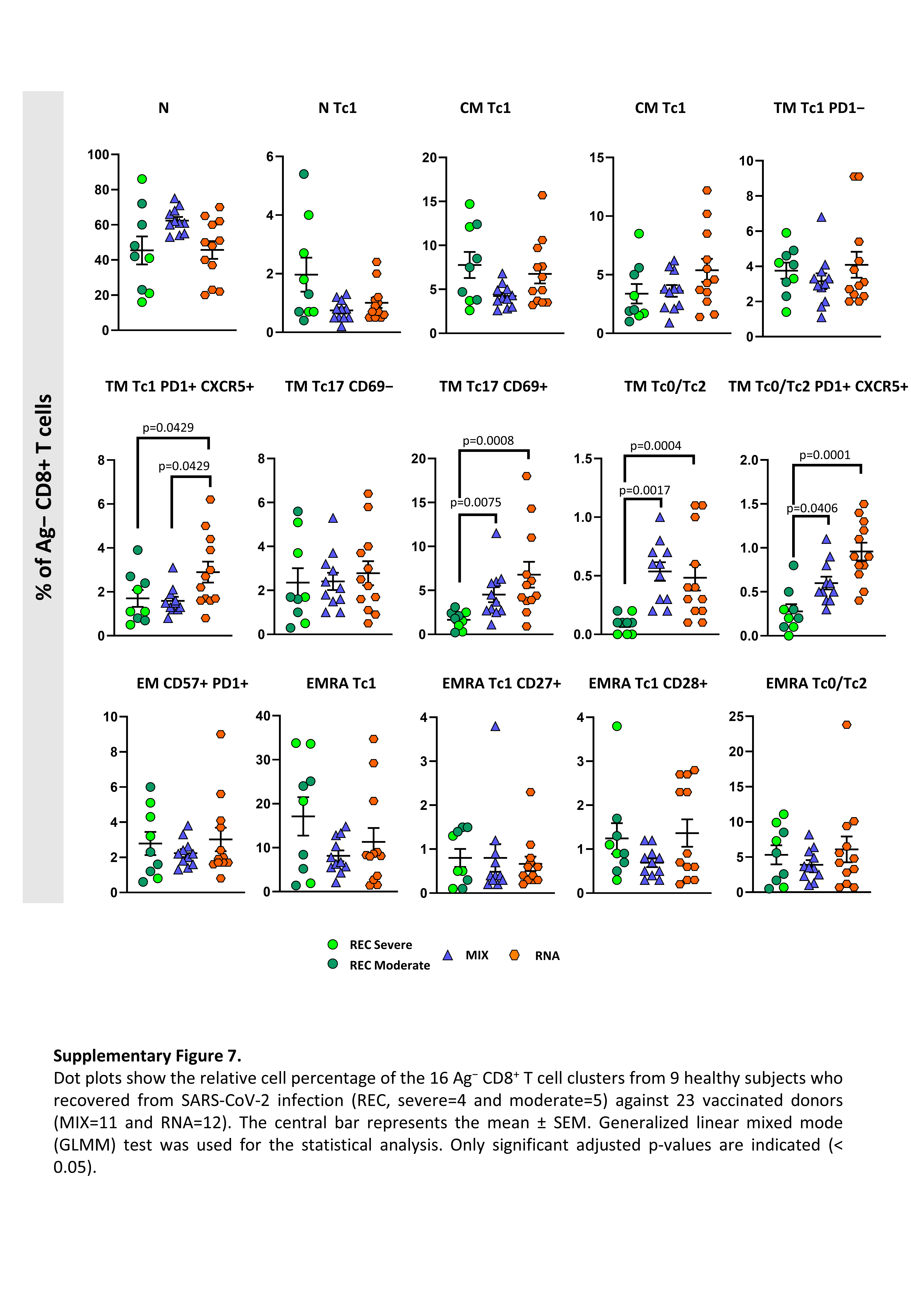

Supplement: Supplementary file 7 [file Image_7.tif]

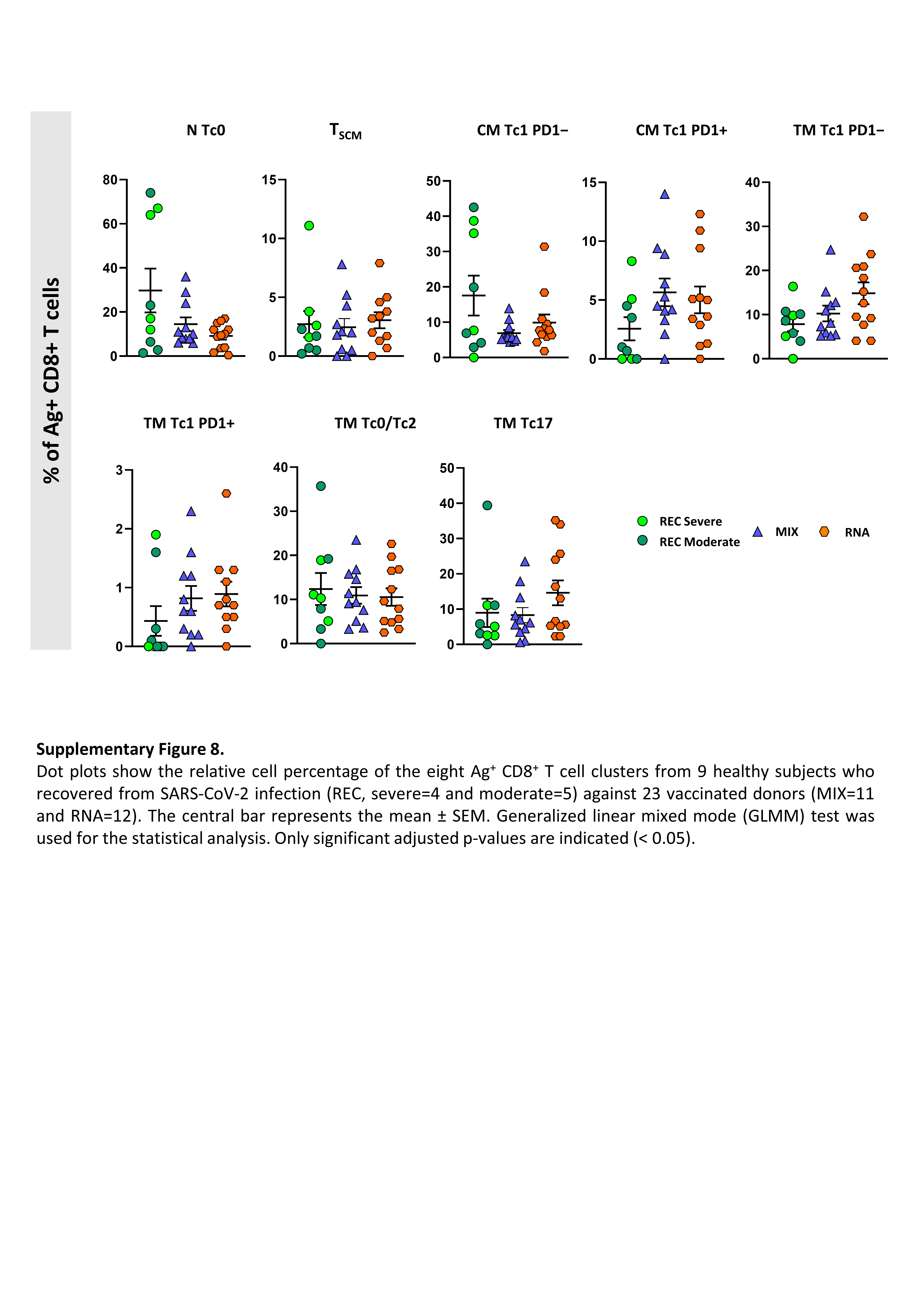

Supplement: Supplementary file 8 [file Image_8.tif]

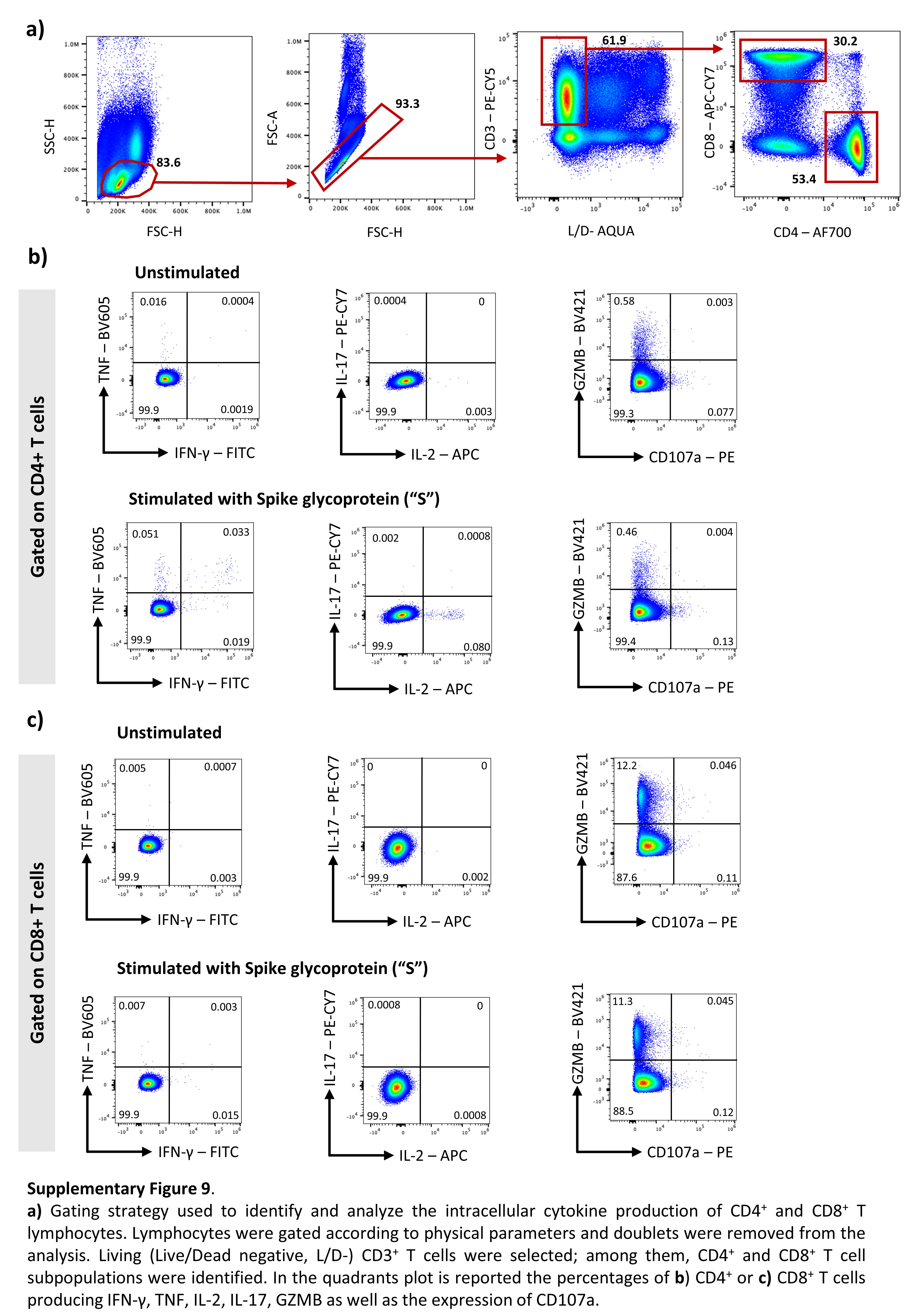

Supplement: Supplementary file 9 [file Image_9.tif]

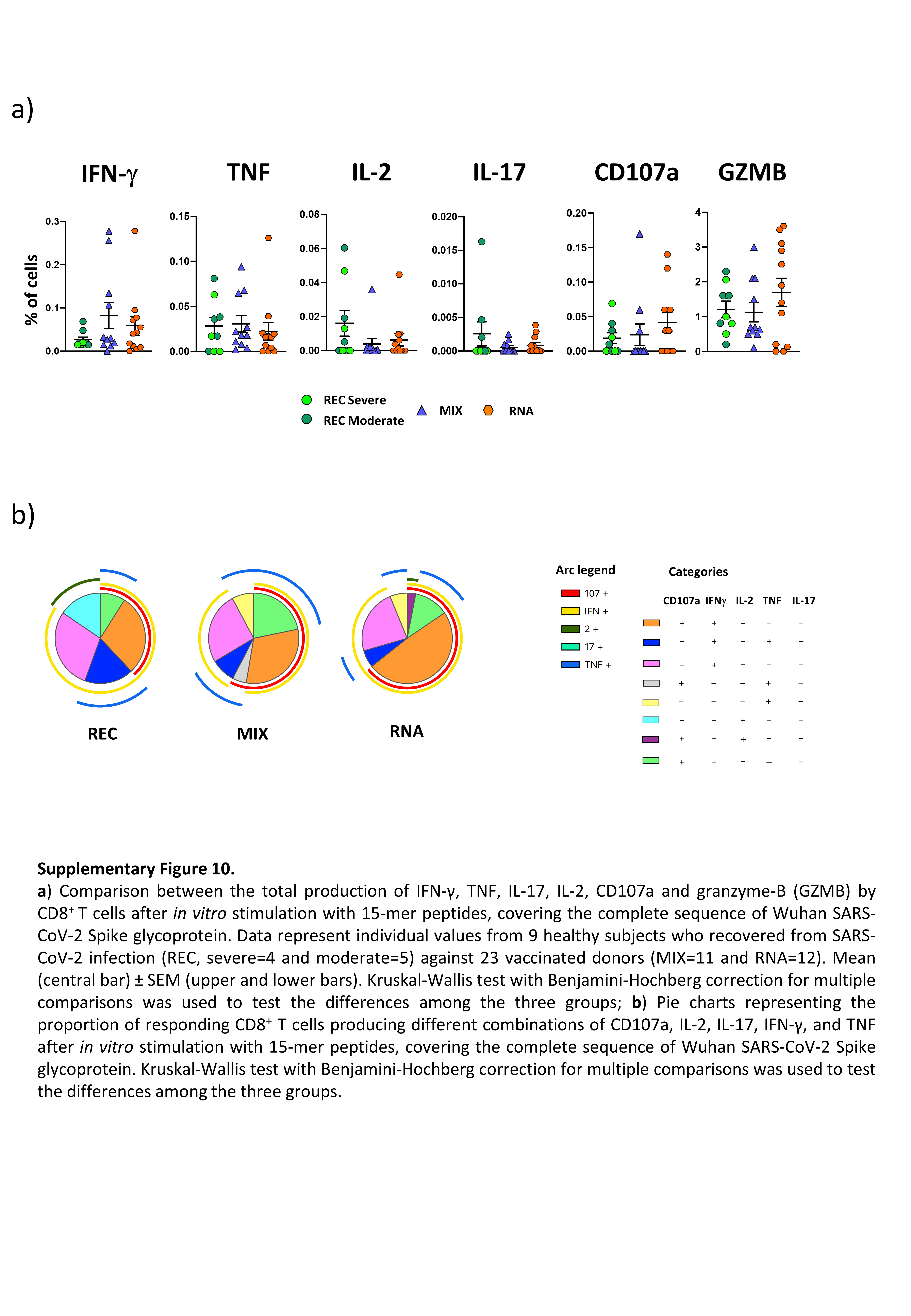

Supplement: Supplementary file 10 [file Image_10.tif]

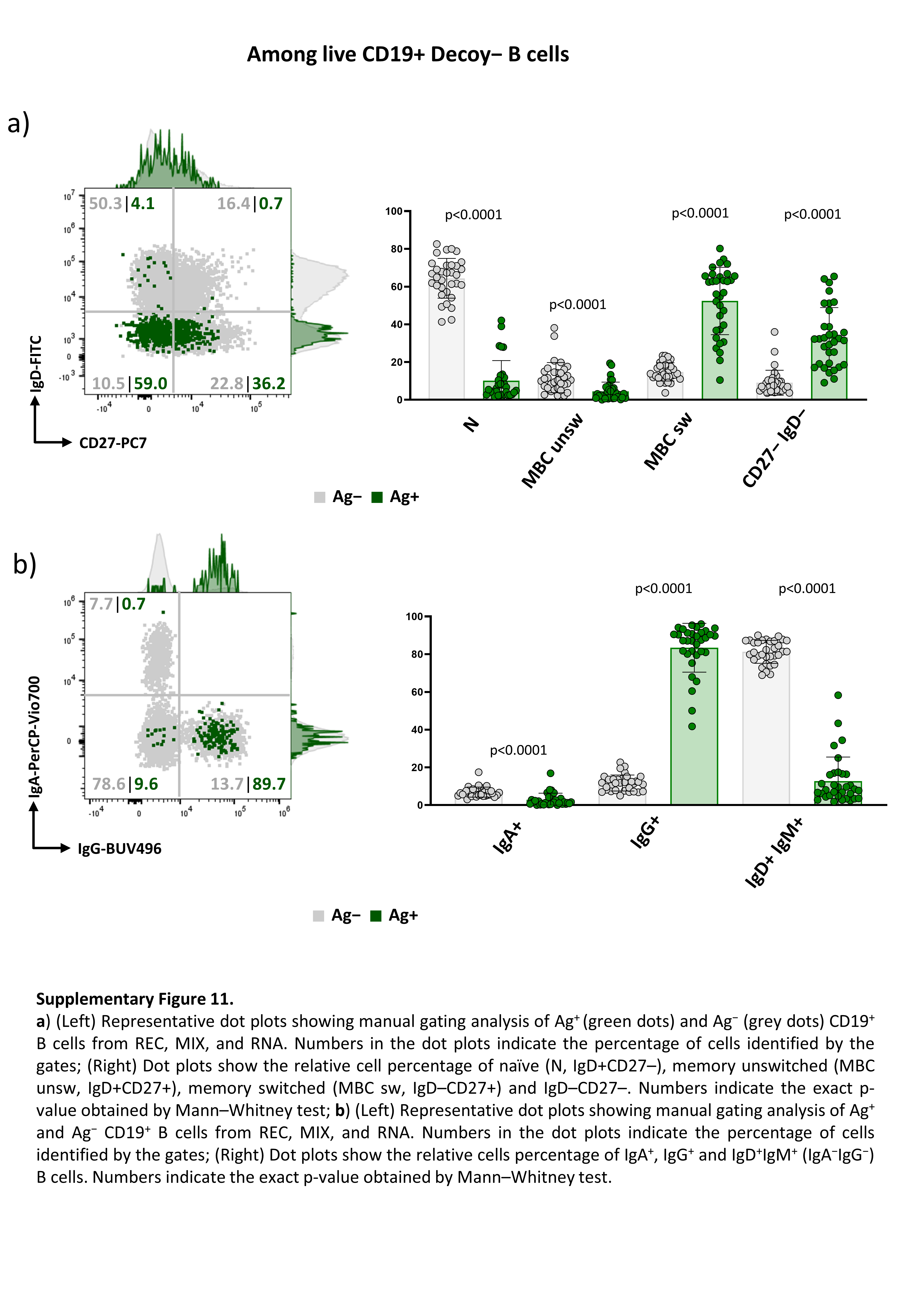

Supplement: Supplementary file 11 [file Image_11.tif]

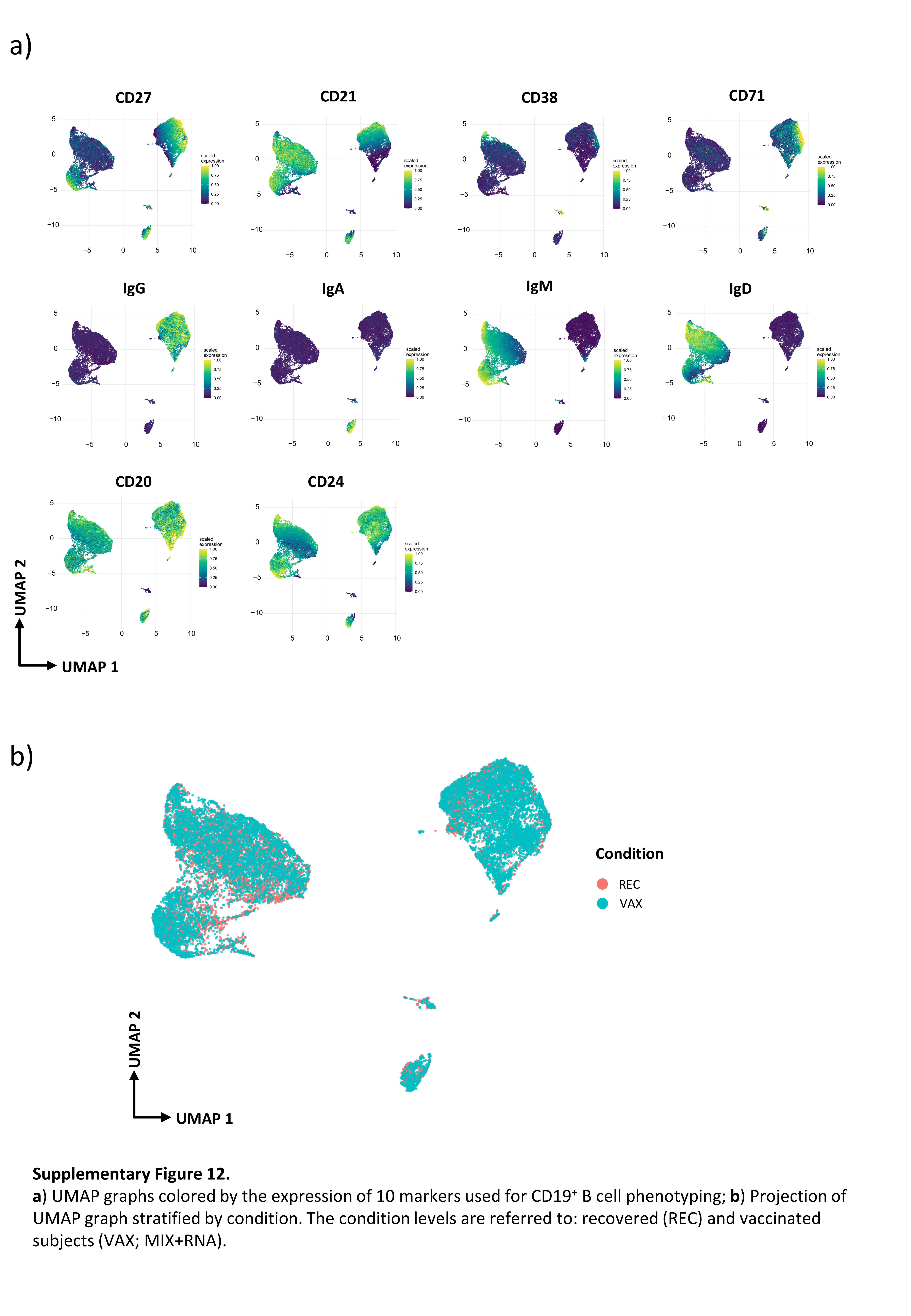

Supplement: Supplementary file 12 [file Image_12.tif]

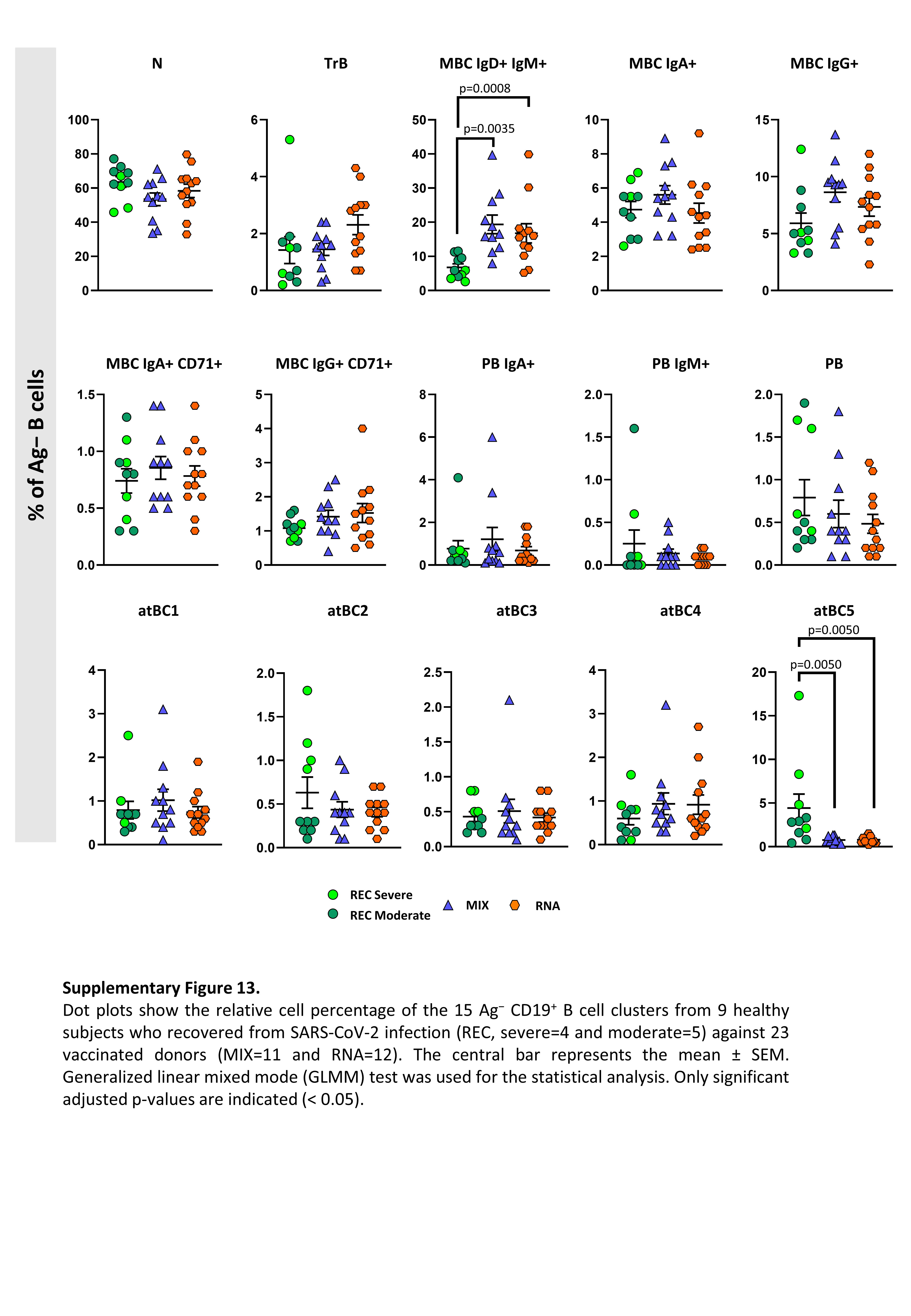

Supplement: Supplementary file 13 [file Image_13.tif]

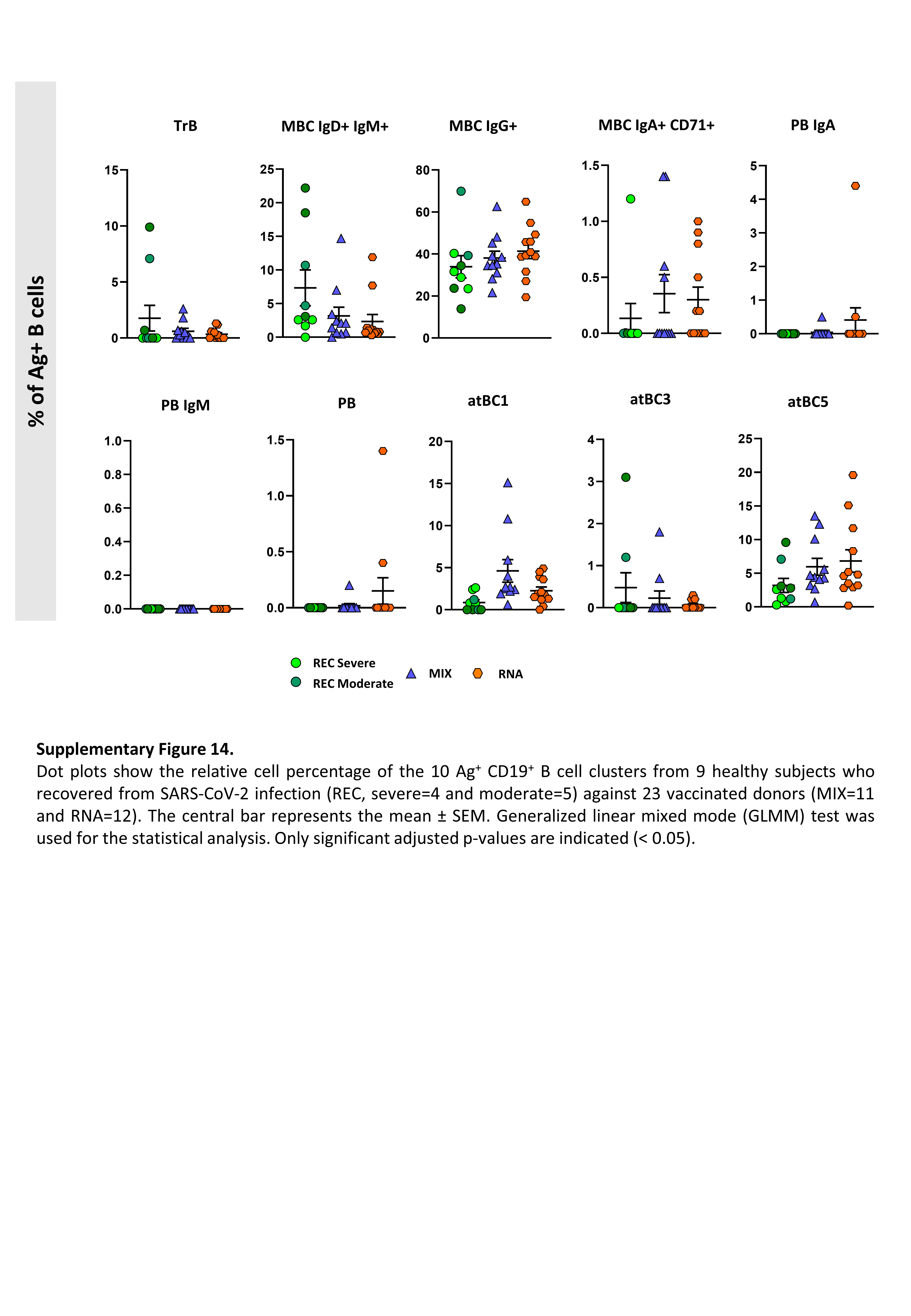

Supplement: Supplementary file 14 [file Image_14.tif]

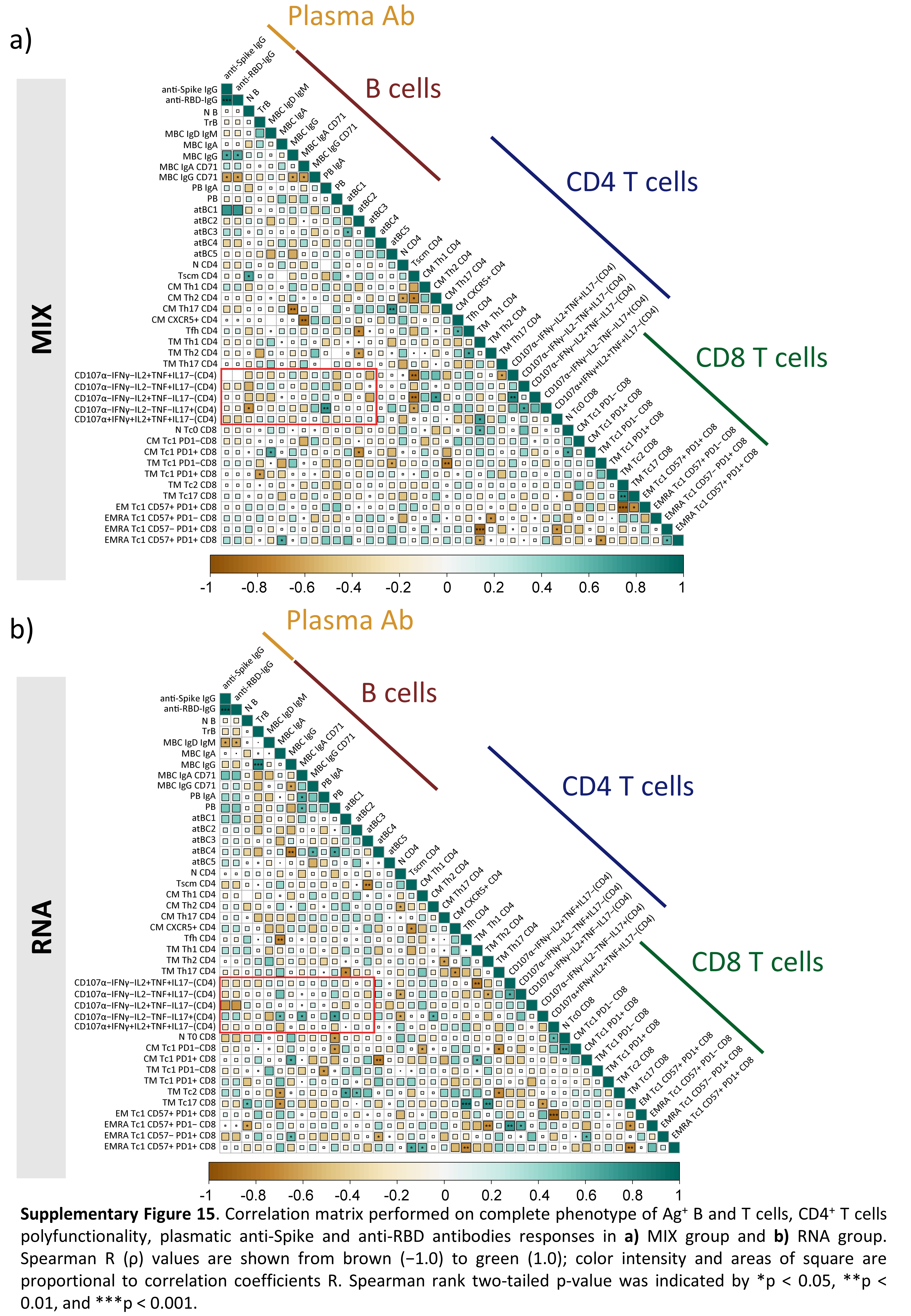

Supplement: Supplementary file 15 [file Image_15.tif]

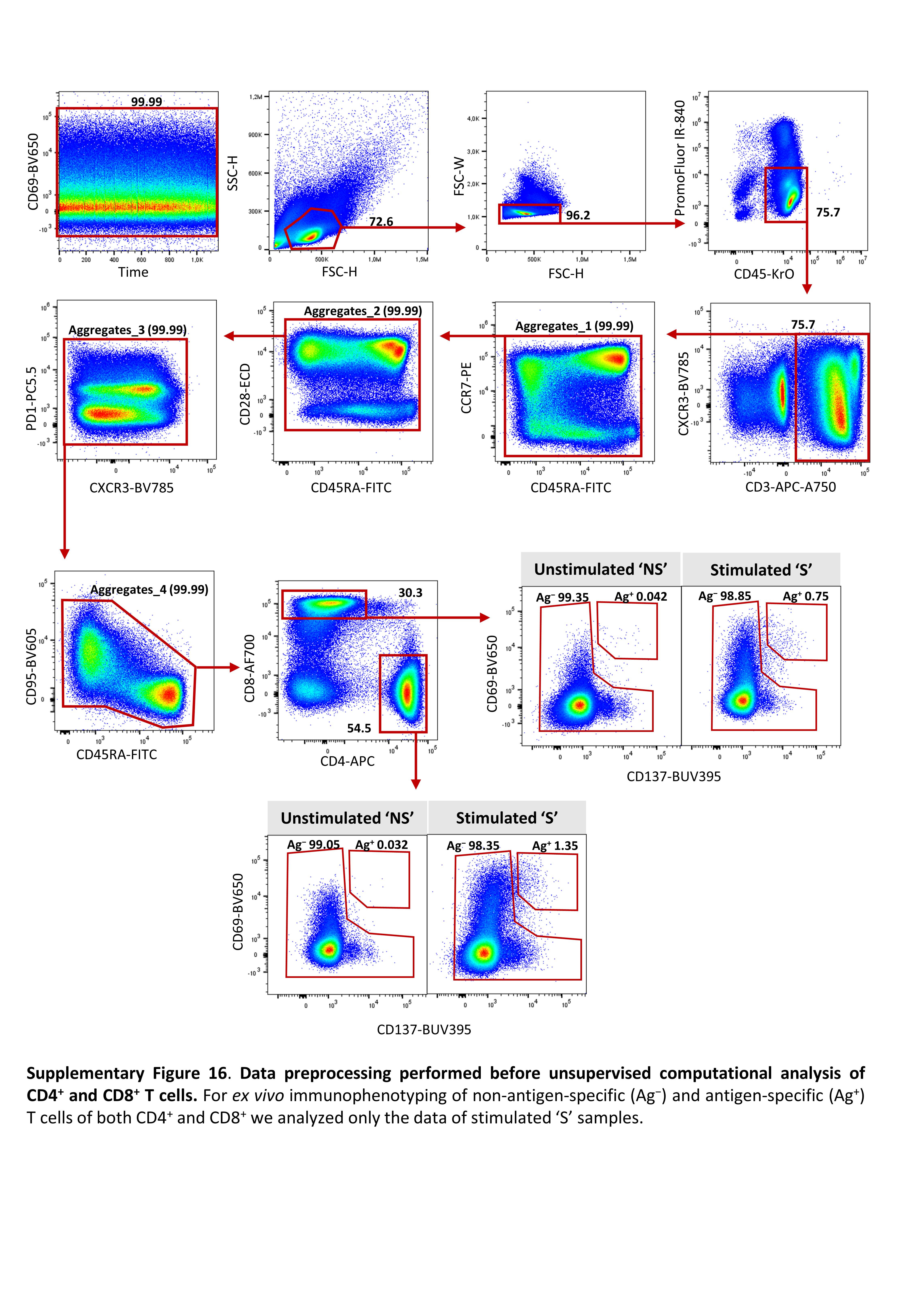

Supplement: Supplementary file 16 [file Image_16.tif]

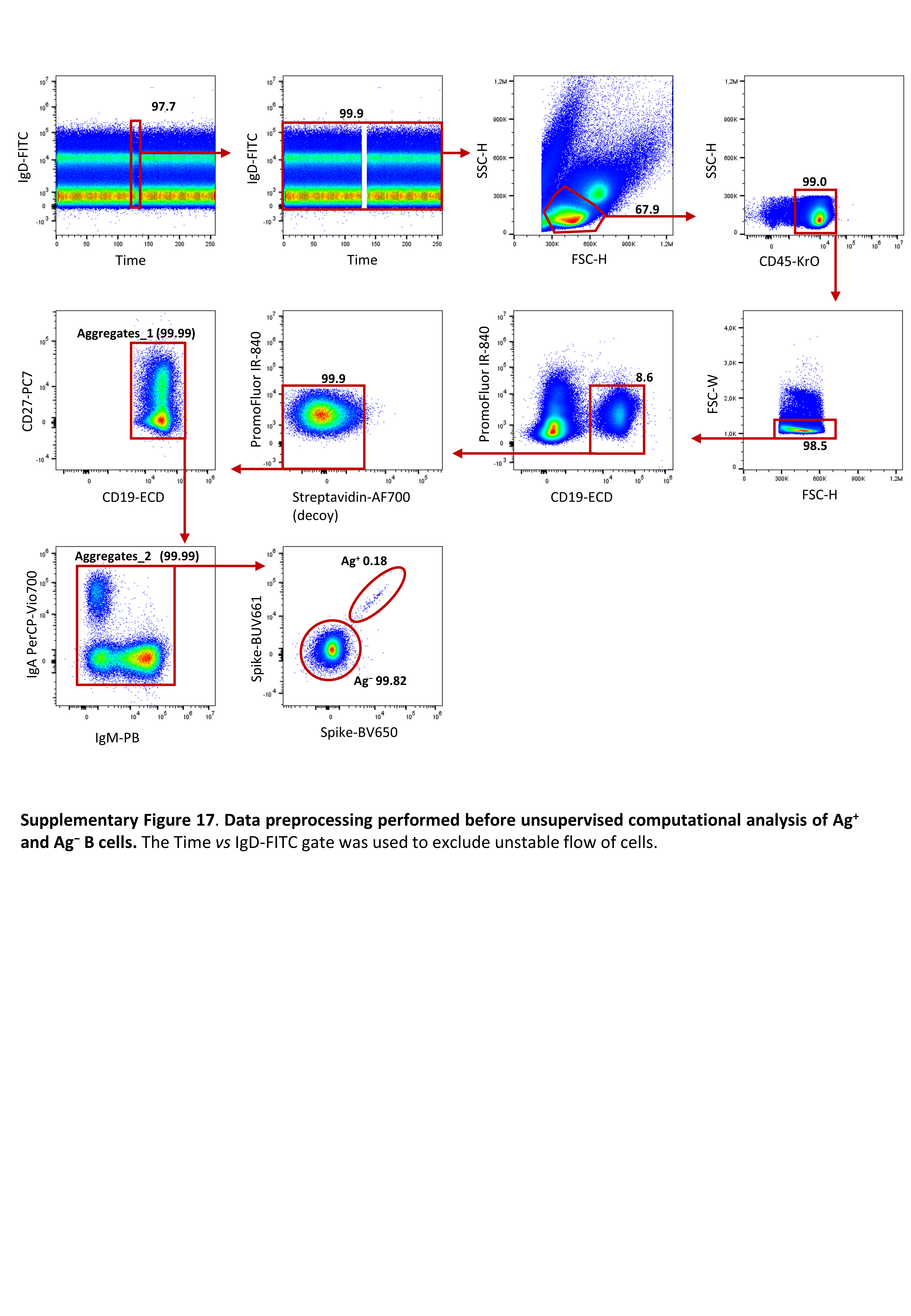

Supplement: Supplementary file 17 [file Image_17.tif]
