# Supplementary material for: Detailed characterization of SARS-CoV-2-specific T and B cells after infection or heterologous vaccination
Source: Front Immunol. 2023 Feb 9;14:1123724. doi: 10.3389/fimmu.2023.1123724 (PMC9947839; doi:10.3389/fimmu.2023.1123724)
Supplement: Supplementary file 18 [file Table_1.docx]

**Supplementary Table 1. List of antibodies used in flow cytometry panels to identify antigen-specific T and B cells.**

| **Target** | **Dye** | **Clone** | **Producer** | **Catalog Number** | **Lot Number** | **Titer (μL)/100μL** | **Panel** |
| --- | --- | --- | --- | --- | --- | --- | --- |
| **PromoFluor840** | Maleimide | N/A | Promocell | PK-PF840-3- 01 |  | 0.3 | AIM assay and B |
| **CD45RA** | FITC | 2H4 | Beckman Coulter (DuraClone IM T) | B53328 |  | - | AIM assay |
| **CCR7** | PE | G043H7 | Beckman Coulter (DuraClone IM T) | B53328 |  | - | AIM assay |
| **CD28** | ECD | CD28.2 | Beckman Coulter (DuraClone IM T) | B53328 |  | - | AIM assay |
| **PD-1** | PC5.5 | PD1.3.5 | Beckman Coulter (DuraClone IM T) | B53328 |  | - | AIM assay |
| **CD27** | PC7 | 1A4.CD27 | Beckman Coulter (DuraClone IM T) | B53328 |  | - | AIM assay |
| **CD4** | APC | 13B8.2 | Beckman Coulter (DuraClone IM T) | B53328 |  | - | AIM assay |
| **CD8** | A700 | B9.11 | Beckman Coulter (DuraClone IM T) | B53328 |  | - | AIM assay |
| **CD3** | APC-A750 | UCHT-1 | Beckman Coulter (DuraClone IM T) | B53328 |  | - | AIM assay |
| **CD57** | Pacific Blue | NC1 | Beckman Coulter (DuraClone IM T) | B53328 |  | - | AIM assay |
| **CD45** | Krome Orange | J33 | Beckman Coulter (DuraClone IM T) | B53328 |  | - | AIM assay |
| **CXCR3** | BV785 | G025H7 | BioLegend | 353738 | B302668 | 1.25 | AIM assay |
| **CCR6** | BUV496 | 11A9 | Becton Dickinson | 612948 | 1114714 | 1.25 | AIM assay |
| **CXCR5** | BUV661 | RF8B2 | Becton Dickinson | 741559 | 1298915 | 0.6 | AIM assay |
| **CD69** | BV650 | FN50 | BioLegend | 310934 | B346313 | 2.5 | AIM assay |
| **CD137** | BUV395 | 4B4-1 | Becton Dickinson | 745737 | 1298922 | 1.25 | AIM assay |
| **CD95** | BV605 | DX2 | BioLegend | 305628 | B344380 | 2.5 | AIM assay |
| **LIVE DEAD** | AQUA | N/A | ThermoFisher | L34966 | 2268307 | 1.25 | ICS |
| **CD3** | PE-Cy5 | UCHT1 | BioLegend | 300410 | B270168 | 0.6 | ICS |
| **CD4** | AF700 | RPA-T4 | BioLegend | 300526 | B336913 | 0.6 | ICS |
| **CD8a** | APC-Cy7 | RPA-T8 | BioLegend | 301016 | B300873 | 0.6 | ICS |
| **IFN-γ** | FITC | B27 | BioLegend | 506504 | B286029 | 2.5 | ICS |
| **IL-2** | APC | MQ1-17H12 | BioLegend | 500310 | B313276 | 2.5 | ICS |
| **Granzyme B** | BV421 | QA18A28 | BioLegend | 396414 | B311965 | 2.5 | ICS |
| **IL-17A** | PE-Cy7 | BL168 | BioLegend | 512315 | B325831 | 3.75 | ICS |
| **TNF** | BV605 | MAb11 | BioLegend | 502936 | B327946 | 3.75 | ICS |
| **CD107a** | PE | H4A3 | BioLegend | 328608 | B321484 | 0.3 | ICS |
| **IgD** | FITC | IA6-2 | Beckman Coulter (DuraClone IM B) | B53318 |  | - | B |
| **CD21** | PE | BL13 | Beckman Coulter (DuraClone IM B) | B53318 |  | - | B |
| **CD19** | ECD | J3-119 | Beckman Coulter (DuraClone IM B) | B53318 |  | - | B |
| **CD27** | PC7 | 1A4CD27 | Beckman Coulter (DuraClone IM B) | B53318 |  | - | B |
| **CD24** | APC | ALB9 | Beckman Coulter (DuraClone IM B) | B53318 |  | - | B |
| **CD38** | APC-A750 | LS198-4-3 | Beckman Coulter (DuraClone IM B) | B53318 |  | - | B |
| **IgM** | Pacific Blue | SA-DA4 | Beckman Coulter (DuraClone IM B) | B53318 |  | - | B |
| **CD45** | Krome Orange | J33 | Beckman Coulter (DuraClone IM B) | B53318 |  | - | B |
| **Streptavidin** | BV650 | - | BioLegend | 405231 | B347044 | 0.3 | B |
| **Streptavidin** | BUV661 | - | Becton Dickinson | 612979 | 1188291 | 0.3 | B |
| **Streptavidin** | AF700 | - | ThermoFisher | S21383 | 2286302 | 0.1 | B |
| **S-protein** | Biotin | DOJH0421071 | R&D | Bt10549 | - | 4.5 | B |
| **IgG** | BUV496 | G18-154 | Becton Dickinson | 741172 | 1341490 | 1.25 | B |
| **IgA** | PerCP-Vio700 | 1S11-8E10 | Miltenyi Biotec | 130-113-478 | 5211109889 | 0.5 | B |
| **CD71** | BUV395 | M-A712 | Becton Dickinson | 743308 | 1298918 | 1.25 | B |
| **CD20** | BV785 | 2H7 | BioLegend | 302356 | B337363 | 0.6 | B |

AIM assay: Activation Induced Marker assay
ICS: Intracellular Cytokine Staining

B: Ag^+^ B cell phenotype
